# Microneedle‐Based Biofuel Cell with MXene/CNT Hybrid Bioanode: Fundamental and Biomedical Application

**DOI:** 10.1002/advs.202516229

**Published:** 2025-09-29

**Authors:** Shoujie Guan, Jingxi Wang, Yang Yang, Xun Zhu, Jianping Zhou, Dingding Ye, Rong Chen, Qinlin Fan, Qiang Liao

**Affiliations:** ^1^ Key Laboratory of Low‐grade Energy Utilization Technologies and Systems Chongqing University Ministry of Education Chongqing 400030 China; ^2^ Institute of Engineering Thermophysics School of Energy and Power Engineering Chongqing University Chongqing 400030 China; ^3^ Department of Orthodontics The Affiliated Hospital of Stomatology Chongqing Medical University Chongqing 400030 China; ^4^ Department of Neurology Second Affiliated Hospital of Army Medical University Chongqing 400030 China

**Keywords:** biofuel cell, microneedle patch, self‐powered, transdermal drug delivery system

## Abstract

Biofuel cells show emerging potential as energy‐supply platform by harvesting bioenergy from human biofluids for next‐generation self‐powered transdermal drug delivery systems. Herein, the study proposes the hydrogel‐based microneedle patch as the drug penetration unit within the corneum to drive the large drug molecules’ delivery. Benefiting from the high porosity and swelling capability, the interstitial fluid can be extracted efficiently to the body surface. One biofuel cell is integrated into the microneedle patch with MXene/carbon nanotube functionalized glucose oxidase bioanode. The transdermal currents of 4–5 µA and open‐circuit potential of 0.28 V are collected in in vivo. The transdermal delivery efficiency and releasing rate of ciprofloxacin and insulin are significantly enhanced in vitro and in vivo models, respectively. The theoretical simulations provide detailed insights into the good electron transport and substrate adsorption, glucose replenishment, and drug delivery under electric field, benefiting the development of transdermal drug delivery systems.

## Graphical abstract

A hybrid microneedle‐based biofuel cell composed of MXene/CNT/glucose oxidase bioanode and platinum/carbon cathode was prepared for wearable transdermal charged drug delivery system. It harvests the subcutaneous interstitial fluid energy and exhibits a stable transdermal current of 4‐6 µA@5 kΩ. With the help of diffusion‐, electric‐ and electroosmotic‐manners, the performance of transdermal delivery of insulin and ciprofloxacin is significantly improved.

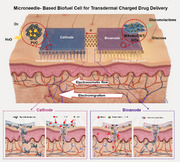

## 1 Introduction

Transdermal drug delivery offers excellent patient compliance as it is noninvasive and can be self‐administered.^[^ ^1^, ^2^ ^]^ It bypasses the first‐pass effect of the liver, which makes it a promising alternative to oral delivery and hypodermic injections.^[^ ^3^, ^4^ ^]^ The first‐generation transdermal drug delivery systems (e.g., metered liquid sprays, gels, and other topical formulations) have a poor drug availability caused by the thick stratum corneum (Thickness: 10–20 µm).^[^ ^5^ ^]^ To deepen the penetration degree, researchers begin to use some external stimulus, such as chemical stimulant, iontophoresis, and non‐cavitational ultrasound, to benefit the drug delivery performance.^[^ ^1^, ^5^, ^6^ ^]^ Among them, iontophoresis represents one of the most prevailing pathways due to its minimal skin irritation and controllable drug delivery kinetics. An energy supply unit should always be proposed to provide an electrical driving force to facilitate drug transport across the stratum corneum.^[^ ^7^, ^8^ ^]^ The common energy supply units for iontophoresis include batteries, capacitors, and biofuel cells (BFCs).^[^ ^6^, ^7^, ^9^, ^10^, ^11^ ^]^ The rechargeable batteries can deliver the power density of hundreds of mW g^−1^ with a large current, but the systems are relatively bulky.^[^ ^12^ ^]^ The BFCs are light and flexible, which can be easily integrated with drug delivery devices and attached to the skin. They usually exhibit the power density of µW cm^−2^ to a few mW cm^−2^, and can better maintain the activity of drugs and biocompatibility, although their current output is still lower than commercial batteries.^[^ ^11^, ^12^ ^]^

BFCs can directly pump the electrons contained in biofluids via an oxidation reaction, such as sweat, blood, and interstitial fluid.^[^ ^13^ ^]^ As the electron acceptor, the ambient oxygen or oxygen contained in the interstitial fluid is typically catalyzed via the oxygen reduction reaction (ORR).^[^ ^14^ ^]^ To trigger the above bio‐reactions, the enzymes such as glucose oxidase (GOx), lactate oxidase (LOx), laccase, and bilirubin oxidase (BOx), are always embedded into the carbonaceous or metallic support as the bioelectrode.^[^ ^12^, ^14^ ^]^ BFCs have shown the emerging potential as a self‐powered platform for drug delivery in vitro. For example, Xiao et al. used the BFCs as a self‐powered platform to control the drug releasing process in vitro, and the releasing performance of three model drugs was evaluated by switching “on” with glucose and dioxygen, and switching “off” at open circuit.^[^ ^15^ ^]^ Nishizawa et al. designed a BFC‐based skin patch (Fructose/O_2_ cell) to generate a transdermal ionic current that facilitated the penetration of small molecules (<≈500 Da) through the dermal barrier in vitro.^[^ ^16^ ^]^ The previous BFC‐based drug delivery systems in vitro were always supplied with sufficient biofuels and oxidizing agent. However, the supply rate of sweat is only 0.2–0.8 µL min^−1^ for BFCs, with the characteristics of fluctuating and intermittent.^[^ ^17^, ^18^ ^]^ Therefore, the lean liquid and unsustainable fuel supply in the practical environment always retards the development of wearable sweat‐based BFCs, leading to the inconsistent and insufficient power output.^[^ ^19^ ^]^ More importantly, when the biofuel cell is integrated into the drug delivery system, it should meet the demands of sufficient and stable voltage/current supply to achieve the continuous operation. Therefore, the robust biofluid and oxygen atmosphere, and well mechanical adaptation with skin are basic needs for the development of BFC‐based systems.^[^ ^20^, ^21^, ^22^ ^]^ Recently, Wang et al. developed a reliable hybrid system combining BFCs with supercapacitor in vivo, which was designed to harvest biochemical energy from the sweat using the BFC for adequate liquid state and store it in the supercapacitor for subsequent use in lean liquid state. Therefore, it could deliver stable output over long charging periods and exhibit favorable cycling ability. Regrettably, the device could only operate at 50 µA for less than 20 min for single discharge, which couldn't meet the long‐term power requirement for drug delivery system.^[^ ^19^, ^23^, ^24^ ^]^ Furthermore, previous works usually ignore the high impedance of the skin (≈10 MΩ), which can reduce the current output of these cells.^[^ ^9^ ^]^ Lastly, iontophoresis is insufficient for the transdermal delivery of large molecules unless combined with other methods to enhance the skin permeability.^[^ ^1^ ^]^

As a third‐generation transdermal drug delivery system, hydrogel‐based microneedle (MN) patch presents a promising strategy for effective drug administration.^[^ ^25^, ^26^ ^]^ The MNs can easily penetrate through the stratum corneum and swell the interstitial fluid quickly, and glucose in the extracted interstitial fluid is used as biofuel of BFCs. They usually have 10–2000 µm in length, a diameter of less than 300 µm, and failure stress of more than 0.2 N needle^−1^, which can be used to penetrate through the stratum corneum.^[^ ^26^, ^27^, ^28^, ^29^, ^30^ ^]^ Along with the swelling process, the drug molecule can be released through the MNs in the diffusion manner. Some trials have been made by combining the biofuel cell with MN patch to accelerate the drug delivery process, and even control the delivery rate using the energy management system.^[^ ^31^ ^]^ For example, Nishizawa et al. used the BFC (Fructose/O_2_ cell) to generate transdermal electroosmotic flow to enhance the transdermal molecular penetration in vitro.^[^ ^9^ ^]^ The device delivered the high transdermal current of 0.2 mA cm^−2^ for 1 hour, which was attributed to the reduced skin resistance to 40–150 kΩ from MN patch, and the high concentration of extraneous biofuel. It used fluorescently labeled dextran (10 kDa) as a model drug and increased the drug concentration from ≈10 µg mL^−1^ (without BFC) to ≈200 µg mL^−1^ (with BFC) after 1 hour. Unfortunately, the biofuel cell used extraneous 0.2 m replenished fructose, and the current density was only tested in vitro for 1 h with continuous decreasing. Liu et al. adopted the glucose from blood or interstitial fluid of diabetic wounds as biofuel.^[^ ^32^ ^]^ When it was used as a power supply of transdermal drug delivery system, it was demonstrated to the limited delivery rate and efficiency with the current of 1 µA. In order to realize the long‐term drug delivery, mass transfer processes within MN patch and BFC need to be further explained and facilitated, *e.g*. electron transfer between bioanode and enzymes, adsorption of bioanode toward biofuel, consumption and replenishment of biofuel, drug delivery from MN patch to dermal layer under the influence of electric field, etc. The previous studies failed to explain the above processes in depth and apply the hybrid system in vivo to evaluate the practical transdermal electrochemical performance and drug delivery performance in detail.

In this work, we design a glucose‐fed microneedle‐based biofuel cell patch (MBFC), which consists of a BFC unit and two MN patch units. Of which, the BFC unit is composed of an MXene/CNT/GOx bioanode and a Pt/C cathode, the glucose from interstitial fluid and oxygen from the air serving as its fuel and oxidizing agent, respectively. The characteristics of morphology, enzyme immobilization, electron transfer, and the power density are studied in detail. Furthermore, the MN patch unit is made of methacryloylated hyaluronic acid (HAMA) hydrogel. Its porosity, swelling capacity, and mechanical property are characterized to evaluate the interstitial fluid extraction capacity and skin penetration ability. After combining BFC with MN patches, we investigate its electrochemical performance both in vitro and in vivo to evaluate the current output stability and open‐circuit potential (OCP). In addition, its transdermal drug delivery rate and efficiency under the electric field of MBFC are assessed. To better understand the internal mass transfer processes, we use density functional theory (DFT) to study electron transfer and biofuel adsorption capacity within MBFC. The numerical simulation model of the MBFC and the dermal layer is constructed to explain the continuous consumption/replenishment of glucose, accumulation and metabolism of gluconolactone (GDL), and transdermal drug delivery.

## 2 Results and Discussion

### 2.1 Design and Mass Transfer Processes of MBFC

**Figure 1:**
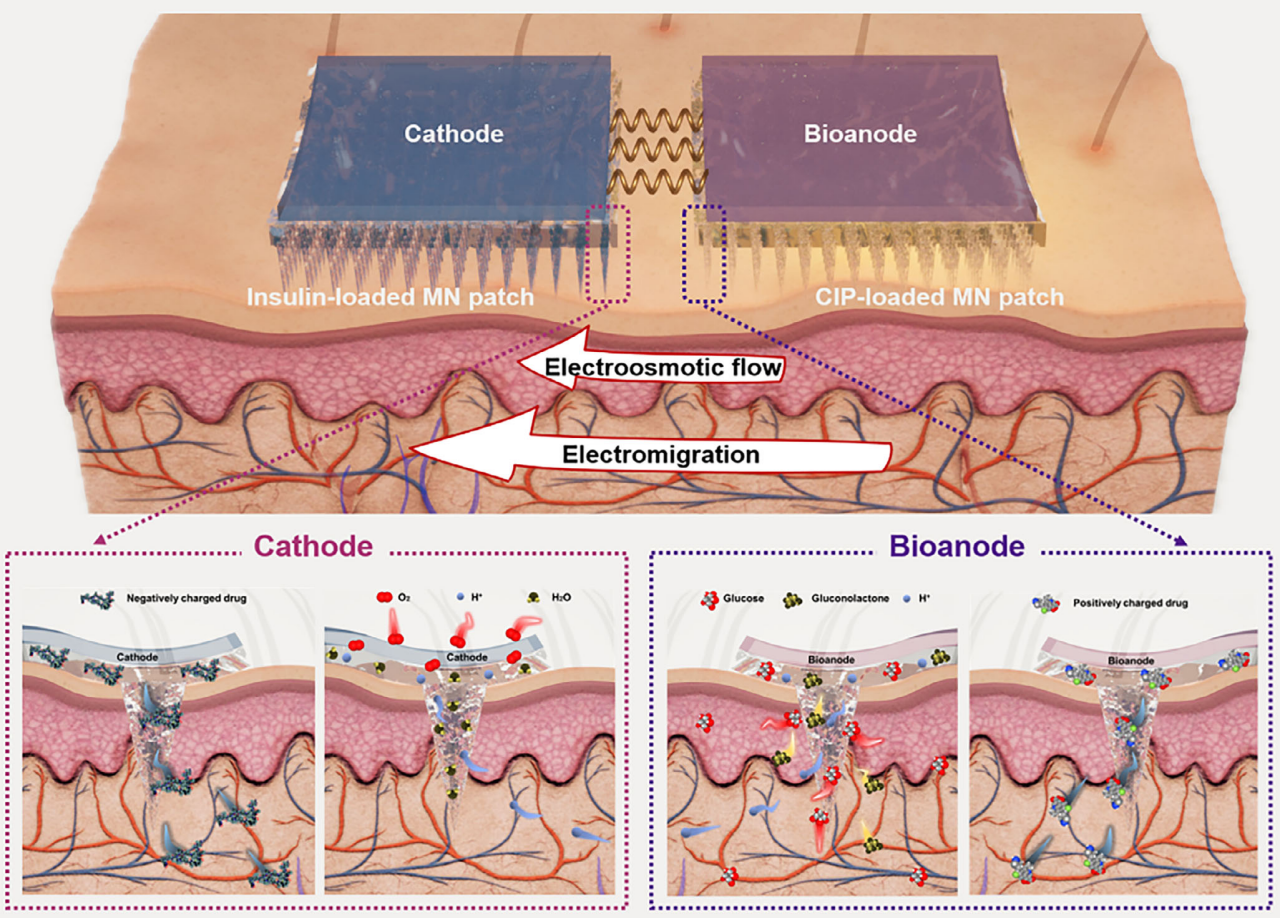
Schematic illustration and internal mass transfer processes of the microneedle‐based biofuel cell. The biofuel cell consists of microneedle patches, Pt/C cathode, and MXene/CNT/GOx bioanode. Glucose and oxygen are sourced from interstitial fluid and ambient air, respectively. The charged drug stored within the microneedle patch is delivered into the dermal layer through diffusion, electric field, and electroosmotic flow manners.

**Figure** **1** illustrates the schematic design of MBFC with MXene/CNT/GOx bioanode, Pt/C cathode, and HAMA hydrogel MN patches, respectively. The bioanode and cathode are affixed to the top surfaces of the drug‐loaded and blank MN patches, respectively, and they are connected via a resistor wire to close the electrical circuit. MN patch penetrates through the stratum corneum to extract the interstitial fluid. At the bioanode, glucose in interstitial fluid diffuses gradually to the electrode surface, and is oxidized with the assistance of MXene/CNT/GOx bioanode to produce GDL, electrons, and hydrogen ions^[^ ^33^ ^]^ The electrons are transferred to the cathode through the external circuit, while hydrogen ions are migrated to cathode through the interstitial fluid, and GDL is transported to dermal layer and metabolized by animal body. At the cathode, oxygen from the air is reduced to form H_2_O, and the required hydrogen ions are supplied by the bioanode reaction.^[^ ^34^ ^]^ The positively or negatively charged drugs stored within MN patch are delivered into the dermal layer through diffusion, electric field, and electroosmotic flow manners.^[^ ^9^ ^]^ Both the electroosmotic flow direction and electromigration direction are from bioanode to cathode. Specifically, the delivery direction of positively charged drug is consistent with the direction of the electric field, while the delivery direction of negatively charged drugs is opposite to the direction of the electric field. For the impact of electroosmotic flow, both delivery directions are consistent with the direction of electroosmotic flow. In this system, the effect of the electric field on the drug is far greater than that of the electroosmotic flow on the drug. Therefore, drugs are more easily delivered when positively charged drugs are positioned interior the bioanode, and negatively charged drugs interior the cathode.

### 2.2 Synthetic Procedure and Characterizations of Bioanode, Cathode, and Biofuel Cell

**Figure 2:**
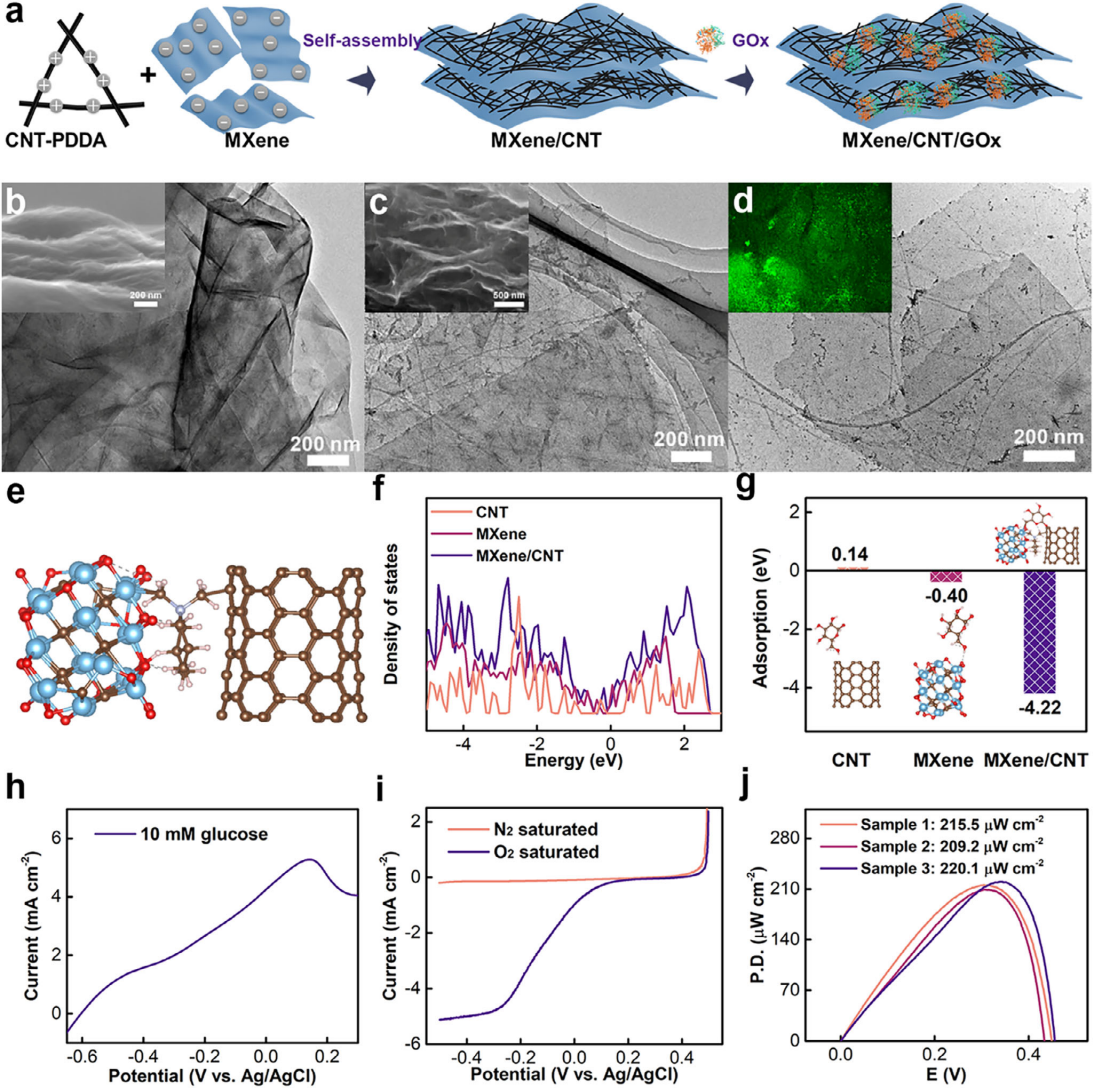
Characterizations and electrochemical measurements of biofuel cell. a) Schematic illustration of the detailed synthetic procedure for MXene/CNT/GOx bioanode. TEM images of MXene (b), MXene/CNT (c) substrates. The inset photos were the corresponding cross‐sectional SEM images. d) The TEM image of MXene/CNT/GOx electrode. The inset photo was the corresponding two dimensioanl (2D) CLSM observation. e) Optimized crystal structure of MXene/CNT. f) Total electron density of states (DOS) for CNT, MXene, and MXene/CNT. g) Adsorption energies of glucose adsorbed on the lattice of CNT, MXene, and MXene/CNT, respectively. Inset photos were the corresponding adsorption‐optimized structures. h) LSV curve of MXene/CNT/GOx bioanode in 0.1 m PBS containing 10 mm glucose. i) LSV curves of Pt/C cathode using a rotating disk electrode with rotation rate of 1600 rpm at 10 mV s^−1^ in 0.1 m PBS under N_2_‐saturated and O_2_‐saturated conditions. j) The power densities of biofuel cells composed of MXene/CNT/GOx bioanode and Pt/C cathode in 0.1 m PBS containing 10 mm glucose under O_2_‐saturated condition using two‐electrode setup. To guarantee the reproducibility, three biofuel cells were fabricated and characterized with LSV procedure.

The bioanode was mainly composed of GOx and MXene/CNT nanocarrier. MXene/CNT has been proven to be able to provide good catalytic microenvironment for LOx due to its large specific surface area, high conductivity, and efficient electron transfer between LOx and nanocarrier.^[^ ^35^ ^]^ Specifically, this bioanode was also prepared using a previously reported electrostatic self‐assembly method, as illustrated in **Figure** **2**a.^[^ ^35^, ^36^ ^]^ First, the positively charged CNT was modified with the cationic polymer poly(diallyldimethylammonium chloride) (PDDA) and combined with negatively charged single‐layer MXene nanosheets to form a three‐dimensional (3D) hierarchical structure.^[^ ^35^, ^37^ ^]^ The variation of Zeta potentials in aqueous solution during the substrate's fabrication process is shown in Figure S1 (Supporting Information). The Zeta potential of CNT was ‐12.6 mV, and changed to +26.3 mV after PDDA modification. The Zeta potential of MXene was measured to ‐18.2 mV. MXene and CNT‐PDDA were bound together to form MXene/CNT with the Zeta potential of ‐38.4 mV through electrostatic self‐assembly. Subsequently, glucose oxidase (GOx) was incorporated into the 3D interlayer structure. Figure 2b and Figure S2a (Supporting Information) displayed the TEM and SEM images of single‐layer MXene substrate, which showed the typical nanosheets’ fold structure. The nanosheets can facilitate the electron transfer rapidly along the horizontal direction of MXene nanosheets. The inset photo of Figure 2b shows its cross‐sectional SEM image, and the aggregation and face‐to‐face self‐restacking of MXene nanosheets were observed due to the strong Van der Waals interaction between adjacent MXene nanosheets.^[^ ^38^ ^]^ This structure lowered the electrochemical active area and GOx loading, as well as deteriorated accessibility to substrate and ions. Figure 2c and Figure S2b (Supporting Information) showed the TEM and SEM images of MXene/CNT substrate, and the single‐walled CNTs were uniformly attached to the surface of MXene nanosheets. The inset photo of Figure 2c proves that the CNTs introduced into adjacent MXene nanosheets and successfully avoided the self‐restacking of MXene. As electrically conductive spacers, CNT with high electrical conductivity allowed the electrons could be transferred rapidly along the vertical direction of MXene nanosheets, and it also shortened the transportation and diffusion distance of substrate and ions.^[^ ^39^ ^]^ As the XRD patterns showed (Figure S3, Supporting Information), the characteristic peak of (002) was observed for both MXene and MXene/CNT, which was related to the layer spacing of MXene. (002) peak of MXene shifted from 6.55° and 5.83°, and the corresponding interlayer spacings increased from 13.5 to 15.0 Å after introducing the CNTs, as calculated using Bragg's equation.^[^ ^38^ ^]^ The result evidenced that the introduction of CNTs increased the neighboring MXene nanosheets distance.

Figure 2d and Figure S2c (Supporting Information) showed the TEM and SEM images of MXene/CNT/GOx, respectively. GOx nanoparticles and their nanoclusters were successfully adsorbed onto the MXene/CNT surface. The XPS spectrum, SEM image, and corresponding EDX maps further validated the presence of Ti, O, C, and N (Originating from GOx) elements, and these elements were uniformly distributed across the surface (Figures S4 and S5a,b, Supporting Information). The GOx sites labeled with fluorescein isothiocyanate (FITC) were clearly visible in the CLSM image (Inset of Figure 2d).^[^ ^40^ ^]^ Furthermore, the immobilization of GOx nanoparticles onto the MXene/CNT surface was achieved via amide bond formation. Specifically, –NH_2_ groups of GOx combined with –COOH groups of MXene/CNT to form the amide bonds. The formation of amide bonds was proved by the high‐resolution XPS spectra and FTIR spectroscopy together (Figures S5c and S6, Supporting Information). The high‐resolution XPS spectrum of the C 1s region revealed five peaks, which were corresponded to the O─C═O bonds (288.7 eV, –COOH groups from MXene/CNT), N─C═O bonds (288.1 eV, Amide carbon), C─O bonds (286.4 eV), C─C bonds (284.8 eV) and C‐Ti bonds (282.0 eV), respectively.^[^ ^41^, ^42^ ^]^ Additionally, the presence of N–H (3440.9 cm^−1^) and C═O groups (1670.3 cm^−1^) was observed on the bioanode surface.^[^ ^41^ ^]^ Both the existence of N─C═O bonds for XPS spectra and the existence of N─H and C ═ O groups for FTIR spectroscopy on bioanode surface proved the presence of amide bonds between enzymes and enzyme nanocarriers.

Figure S7 (Supporting Information) explained the catalytic reaction process of MXene/CNT/GOx for BFCs. In this system, GOx was used as a catalyst to trigger the oxidation reaction of glucose, MXene/CNT as an enzyme nanocarrier to immobilize the GOx and transfer electrons from GOx, 1,4‐NQ and Buckypaper were used as an electronic mediator and flexible current collector, respectively. The catalytic process can be divided into glucose adsorption, catalytic reaction, and electron transfer. Specifically, MXene/CNT/GOx absorbs glucose from the biofluids. Then, glucose is oxidized to produce gluconolactone and electron under the catalytic action of GOx. This electron is located in the active center of the non‐conductive enzyme. Finally, the electron located in the active center is transferred through direct electron transfer provided by MXene/CNT and mediated electron transfer provided by 1,4‐NQ. These electrons are collected by MXene/CNT and then transferred to Buckypaper.

To elucidate the significance of combining MXene, CNT, and PDDA, the crystal structure of MXene/CNT was modeled (Figure 2e). The crystal structure models of CNT and MXene were connected by PDDA to simulate the MXene/CNT composite.^[^ ^36^ ^]^ Figure 2f,g characterized its electron transfer properties and biofuel adsorption capacity, respectively. To further explore these properties, theoretical calculations based on DFT were performed. Figure S8 (Supporting Information) displayed the optimized crystal structures of MXene and CNT. To reflect the electron filling and conductivity properties, the total electron density of states (DOS) for MXene/CNT, MXene, and CNT was summarized in Figure 2f. MXene/CNT exhibited a greater availability of electron states near the Fermi level compared to pristine MXene and CNT, which could benefit the electron excitation. The positively‐charged quaternary ammonium group of PDDA introduced the additional electronic states. Besides, the electron density of state for these materials of P‐orbital was shown in Figure S9 (Supporting Information), which had the same tendency with DOS results. The pDOS peak density of MXene/CNT was significantly higher than that of MXene and CNT. Especially near the Fermi level, it exhibited a dense distribution of electronic states. In addition, the DOS and pDOS of MXene/CNT showed the largest integral area and highest peak density, which might enhance the biofuel adsorption performance. DOS and pDOS of CNT showed the smallest integral area and lowest peak density, which might indicate its limited adsorption capacity for biofuel.

Figure 2g evaluated the adsorption capacity of the materials towards the biofuel molecules (e.g., glucose), and the inset image of Figure 2g showed the corresponding optimized adsorption models. The adsorption energies for CNT, MXene, and MXene/CNT were calculated to 0.14, −0.40, and −4.22 eV, respectively. According to the adsorption energy ranges (i.e., >0 and <0), glucose was classified as being hardly adsorbed and easily adsorbed, and the more negative numerical results tended to be stronger adsorption capacity.^[^ ^43^ ^]^ Among these materials, MXene/CNT exhibited the strongest adsorption capacity for glucose, which ensured efficient delivery of glucose to the bioanode interface and enhanced the catalytic process.

The good conductivity and adsorption performance of MXene/CNT were attributed to introducing PDDA, which could introduce the additional electronic and optimize the electronic energy level distribution, as well as strengthen the interaction between MXene and CNT. In order to prove the above viewpoint, a new crystal structure MXene/CNT was constructed as shown in Figure S10 (Supporting Information), and crystal structure models of CNT and MXene were directly connected to label as MXene‐CNT. The previous MXene/CNT model was labeled as MXene‐PDDA‐CNT. The DOS and pDOS of MXene‐PDDA‐CNT and MXene‐CNT were shown in Figure S11 (Supporting Information), which demonstrated the MXene‐PDDA‐CNT had a greater availability of electron states near the Fermi level. As shown in Figure S12 (Supporting Information), the MXene‐PDDA‐CNT model also showed the stronger adsorption capacity for glucose compared to MXene‐CNT model (‐1.55 eV). The d‐band centers were further illustrated in Figure S13 (Supporting Information), which was corresponding to the adsorption activity and was used to study the relationship between electronic structure characteristics and adsorption performance. D‐band centers of MXene‐PDDA‐CNT, MXene‐CNT, and MXene were located in ≈‐1.0, ‐1.2, and ‐2.1 eV, respectively. D‐band center of MXene‐PDDA‐CNT was much closer to the Fermi level than that of MXene‐CNT and MXene, which demonstrated PDDA could optimize the electronic energy level distribution. Hence, MXene‐PDDA‐CNT had better adsorption performance.

Nevertheless, the DFT calculations were still hard to study the electronic structure and adsorption simulations of bioanode after adding GOx, due to its numerous limitations in computing biological macromolecule. Hence, the GOx was excluded, and the conductivity of distinct nanocarriers and their adsorption capacity for glucose were the focus of attention in order to understand the reasons for their distinct performance. The Bader charge results before and after introducing PDDA are shown in Table S1 (Supporting Information). PDDA monomer had the positive charged C atoms, negative charged H atoms, and negative charged N atoms. The pristine MXene/CNT without monomer had Ti atoms with positive charge, C atoms with negative charge, and O atoms with negative charge. When PDDA was introduced between MXene and CNT, surface negative charge of C atoms and O atoms of MXene/CNT reduced, Ti atoms surface of MXene/CNT, C atoms and H atoms of PDDA monomer obtained the electrons from them. Hence, PDDA was activated, and the interaction between MXene and CNT was strengthened, which improved the conductivity and adsorption performance.

The Pt/C cathode was prepared using a spraying technique to deposit commercial Pt/C onto Buckypaper, and its mass loading of Pt/C was controlled to 1 mg cm^−2^. The Pt/C nanoparticles were uniformly distributed with the diameter of ≈80 nm (Figure S14, Supporting Information) on the surface of the buckypaper. It ensured the high oxygen reduction reaction (ORR) properties.

A conventional three‐electrode system was employed to investigate the glucose oxidation properties of the bioanode and the ORR properties of the cathode. As shown in Figure 2h, the linear sweep voltammetry (LSV) curve of MXene/CNT/GOx in 10 mm glucose solution displayed two distinct oxidation peaks at ≈‐0.50 and 0.14 V vs. Ag/AgCl, compared to that of MXene/CNT (Figure S15, Supporting Information).^[^ ^44^, ^45^, ^46^ ^]^ The two peaks were attributed to efficient direct electron transfer between MXene/CNT and GOx, and mediated electron transfer via 1,4‐naphthoquinone (1,4‐NQ), respectively.^[^ ^33^, ^46^ ^]^ Additionally, LSV curves of the commercial Pt/C cathode were collected under N_2_‐saturated and O_2_‐saturated conditions using a rotating disk electrode (RDE), as shown in Figure 2i. The onset potential (*E_onset_*) was determined to 0.16 V vs. Ag/AgCl, with the maximum reduction current density of 4.93 mA cm^−2^ at ‐0.5 V after subtracting the background current. The internal resistance of bioanode and cathode was 33 and 20 Ω according to the Nyquist plots (Figure S16, Supporting Information), respectively. The low charge‐transfer resistance ensured the rapid electron and charge transfer rate for BFC. It was worth noting that the internal resistance of MXene/CNT increased from 6 to 33 Ω after introducing the non‐conductive GOx. Nyquist plot of MXene/CNT/GOx electrode showed a semicircle with a much larger diameter compared with that of MXene/CNT. It proved that GOx also increased the charge‐transfer resistance for bioanode, which was consistent with the previous literatures.^[^ ^33^, ^41^ ^]^ Nevertheless, the proposed bioanode still maintained the appreciable impedance compared to these literatures.

To evaluate the electrochemical performance of the BFC constructed from these electrodes, a conventional two‐electrode system was used. As shown in Figure 2j and Figure S17 (Supporting Information), the internal resistance of the BFC was approximately 17 Ω, the maximum power density and OCP were measured to be ≈210 µW cm^−2^ and 0.45 V, respectively. These results highlighted the excellent power‐generation capabilities of the BFC, outperforming previously reported glucose‐based BFCs, as summarized in **Table** **1**.

**Table 1:** The performance of glucose‐based BFC reported in the literatures.

Enzyme‐carrier	Anode/Cathode	Conc. [mm]	OCP [V]	Power density [µW cm^−2^]	Refs.
Buckypaper	GDH/Lac	180	0.3	20.8	E. Katz^[^ ^47^ ^]^
CNT	GDH/Lac	60	0.53	30	E. Katz^[^ ^48^ ^]^
PANI/TPU	GOx/Lac	10	0.05	‐	Y. Z. Long^[^ ^49^ ^]^
Rubber fiber	GOx/BOx	7	0.58	36.6	J. Kim^[^ ^50^ ^]^
AuNPs	CDH/BOx	2.5	0.65	7	V. Andoralov^[^ ^51^ ^]^
Polymers	GOx/BOx	10	0.377	1.35	X. Xiao^[^ ^52^ ^]^
CNT fiber	GOx/Pt	6	0.4	32.6	H. Peng^[^ ^53^ ^]^
TPU/CNT	GOx/Pt	5	0.575	57	Y. Yang^[^ ^41^ ^]^
CNT	GOx/Pt	5	0.46	10	J. Wang^[^ ^54^ ^]^
**MXene/CNT**	**GOx/Pt**	**10**	**0.45**	**210**	**This work**

### 2.3 Characterizations of HAMA Microneedle Patch

**Figure 3:**
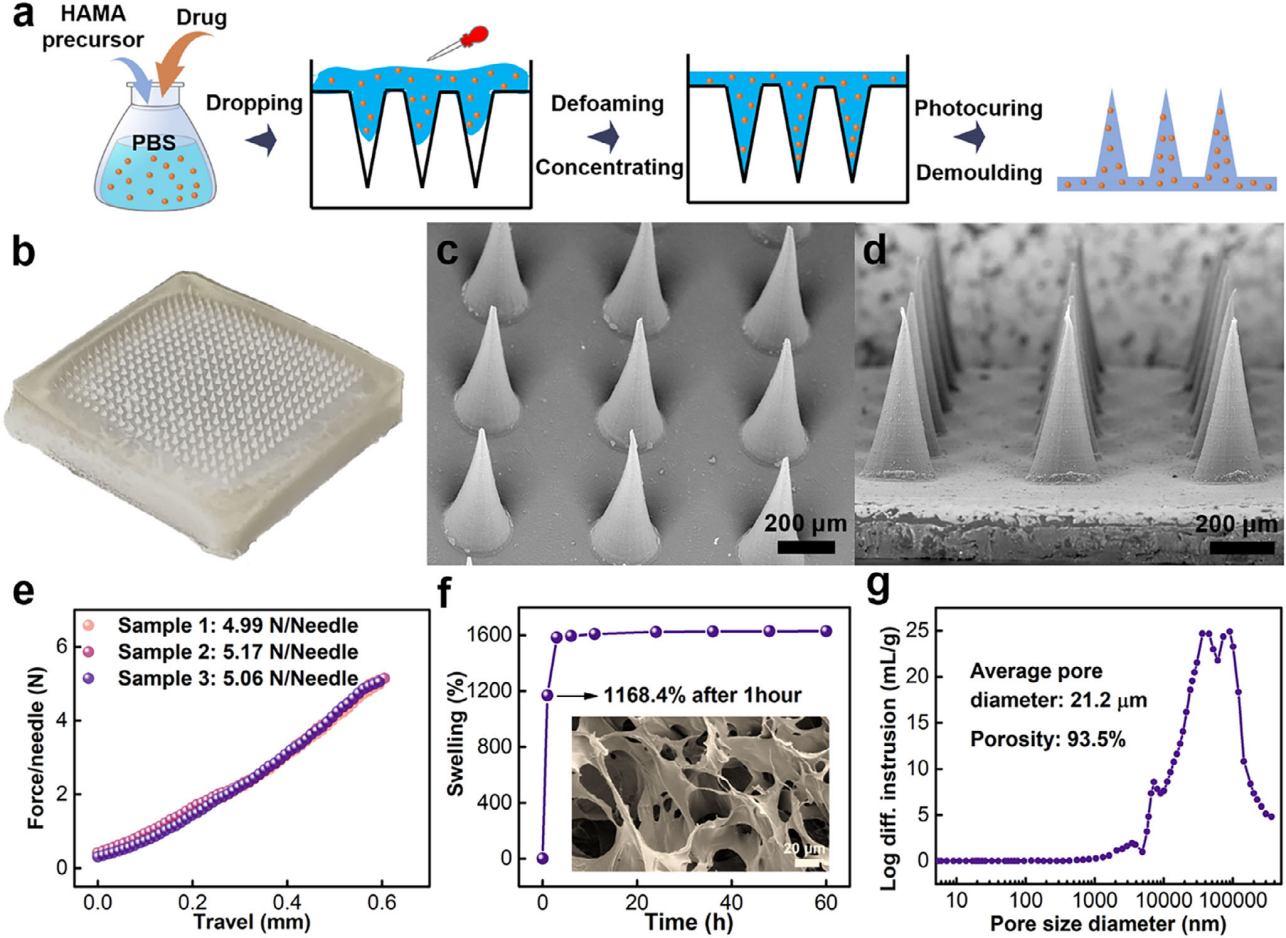
Characterizations of microneedle patch. a) Schematic illustration of the detailed synthetic procedure for microneedle patch. b–d) The photo (b), SEM image (c), and corresponding cross‐sectional SEM image (d) of microneedle patch. e) Mechanical behavior diagram of microneedle patch under vertical force (n = 3). f) The swelling property of microneedle patch in 0.1 m PBS. The inset was the SEM image of HAMA hydrogel after swelling. g) Average pore diameter and porosity of HAMA microneedle patch after swelling.

HAMA hydrogel MN patch was fabricated using a template method, as illustrated in **Figure** **3**a.^[^ ^55^ ^]^ Specifically, a mixture solution of the HAMA precursor and the drug was dispensed into a polydimethylsiloxane (PDMS) mold, and then defoamed and concentrated until drying. Subsequently, the HAMA precursor was photocured to enhance its mechanical properties, and the resulting MN patch was carefully detached from the mold. The MBFC was finally assembled by integrating the bioanode and cathode onto the drug‐loaded and blank MNs patches, respectively. A representative and well‐defined HAMA MN patch was fabricated into a 20 × 20 array, as shown in Figure 3b. Each conical needle had a diameter of approximately 220 µm and a height of about 600 µm, as the SEM images showed (Figure 3c,d). The inter‐tip distance between adjacent MNs was around 500 µm and the substrate of the patch had a thickness of approximately 140 µm. In terms of the mechanical property, the previous studies reported the failure stress of individual needles to 0.2–0.3 N needle^−1^, which was sufficient for effective skin penetration.^[^ ^56^ ^]^ Notably, the failure stress of the HAMA MN patch in our study was measured to 5 N needle^−1^, significantly surpassing with the previous literature. This enhanced mechanical performance could be attributed to the high degree of dryness and the additional photo‐crosslinking step.^[^ ^57^, ^58^ ^]^ Furthermore, the patch demonstrated excellent morphological stability without noticeable changes after being stored in air for six months.

The swelling property of this patch played a critical role in evaluating its biofluid extraction capability. As shown in Figure 3f, the swelling ratio of the patch reached an impressive value of 1168.4% after 1 hour and stabilized at equilibrium after 3 hours. Compared to polyvinyl alcohol‐based hydrogel (45%, 1.5 h), polyacrylamide‐based hydrogel (1250%, over 48 h), and polyacrylic acid‐based hydrogel (800%, 48 h), HAMA hydrogel showed a significantly higher swelling ratio at equilibrium and rapider swelling rate.^[^ ^59^, ^60^, ^61^ ^]^ Furthermore, the patch exhibited numerous macropores with an average diameter of 21.2 µm and porosity of up to 93.5%, as illustrated in the inset photo of Figure 3f,g. The combination of large pore diameter and high porosity significantly enhanced the swelling property, which could facilitate the biofluid extraction and drug penetration processes.^[^ ^26^ ^]^

### 2.4 Transdermal Electrochemical Performance and Drug Delivery In Vitro

**Figure 4:**
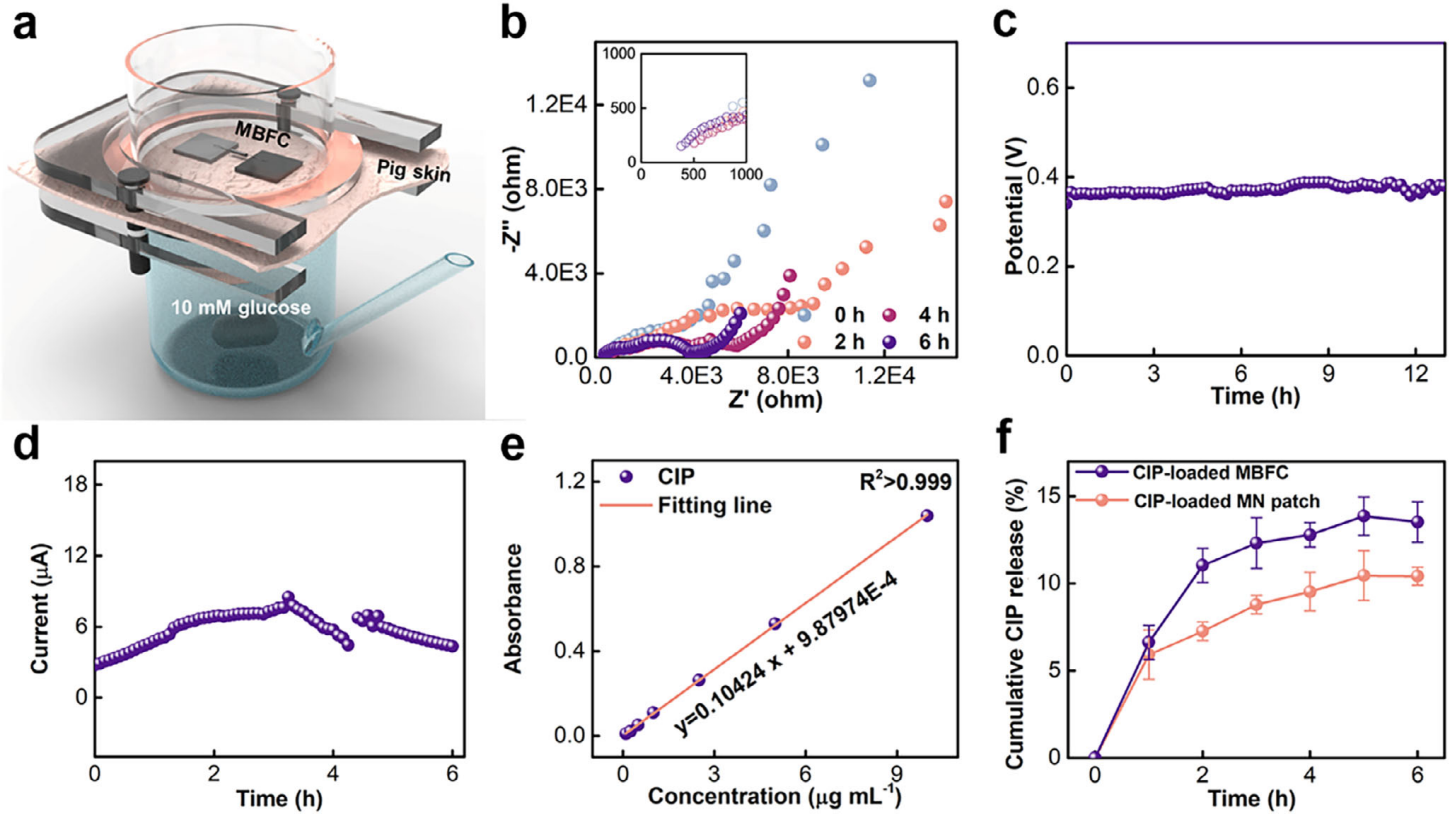
In vitro transdermal electrochemical performance and drug delivery. a) Schematic illustration of Franz Diffusion Cell. b–d) Nyquist plots at different times (b), open‐circuit potential over 12 h (c), and transdermal current @5000 Ω resistor over 6 h (d) of MBFC in Franz Diffusion Cell containing 10 mm glucose in 0.1 m PBS. e) Calibration curve showing the linear relationship between CIP concentration and absorbance in 0.1 m PBS. f) Cumulative CIP delivery profiles using CIP‐loaded MBFC and CIP‐loaded MN patch (n = 3).

To simulate the transdermal performance, a standard Franz Diffusion Cell (FDC) system was employed.^[^ ^62^ ^]^ As shown in **Figures** **4**a and S18 (Supporting Information), Bama pig skin equipped with the MBFC was positioned flat on simulated interstitial fluid to mimic transdermal electricity generation and drug delivery. Of which, the simulated interstitial fluid was 10 mm glucose in 0.1 m PBS, and the Bama pig skin was used due to its similar properties to human skin, and has been widely used in in vitro transdermal experiments.^[^ ^62^ ^]^ Figure 4b illustrated the impedance of the MBFC with the initial internal resistance of ≈400 Ω. Subsequently, the impedance was decreased once the patch was swelled and the electrode was infiltrated. The OCP of MBFC could be stabilized at ≈0.4 V and remained steady for at least 12 hours. When connected to an external resistance, the current was ≈6 µA@5 kΩ for 6 hours. As shown in Figure 4d, the current initially increased and then decreased. It was easily comprehended because the decrease of impedance and increase of glucose concentration on the bioanode surface led to an risen of current initially. With the glucose consumed in the solution, the glucose concentration near the bioanode within the MN patch decreased, resulting in a reduction in current.

To simulate the drug delivery performance in vitro, the CIP with positive charge was selected as the validated platform.^[^ ^61^ ^]^ The Zeta potential of its aqueous solution was measured to +8.0 mV, which proved the CIP molecules surface carried positive charges (Figure S19, Supporting Information). It showed a strong linear relationship between CIP concentration and absorbance, and the characteristic peak at 270.5 nm was unrelated with the existence of glucose or GDL (Figure S20, Supporting Information; Figure 4e). As shown in Figure 4f, the cumulative CIP delivery was evaluated over 6 hours. During the first hour, the cumulative delivery of CIP using the CIP‐loaded MN patch and the CIP‐loaded MBFC was comparable, likely due to the initially high impedance of the MBFC. However, the CIP‐loaded MBFC exhibited a faster delivery rate and efficiency compared to the CIP‐loaded MN patch over the next four hours, which was correlated with the electric field of MBFC. By the fifth hour, CIP delivery into the pig skin decreased. Overall, the CIP‐loaded MBFC achieved a cumulative CIP delivery of 13.5%, which represented an approximately 30% increase compared to the CIP‐loaded MN patch (Cumulative CIP delivery of 10.4%).

### 2.5 In Vivo Transdermal Electrochemical Performance and Drug Delivery

**Figure 5:**
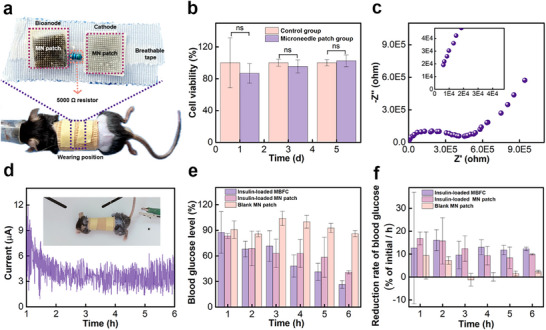
In vivo transdermal electrochemical performance and drug delivery. a) The photo of the mice wearing MBFC. b) The CCK‐8 analysis (n = 6) of microneedle patch. c,d) Nyquist plot for 1 hour (c) and real‐time transdermal current curve for 6 hours (Equipped with 5000 Ω resistor) (d) of MBFC in vivo. e,f) Blood glucose levels (e) and reduction rates of blood glucose (f) in mice wearing different patches (n = 3).

**Figure** **5** demonstrated the effectiveness in vivo operation of the MBFC loaded into a large‐molecule negatively charged drug. Insulin, serving as the negatively charged drug, was loaded into the MN patch near the cathode.^[^ ^4^ ^]^ To evaluate its transdermal electrochemical performance and delivery efficiency, a model of insulin‐deficient diabetic mice wearing the MBFC was established. Its glucose levels were monitored as an indicator of insulin delivery rate and efficiency.^[^ ^3^ ^]^ Figure 5a presented an image of a mice wearing the MBFC. The use of breathable tape and a bandage allowed the MBFC to be stably attached to the skin surface and ensured sufficient oxygen supply to the BFC unit. The mice maintained good physical and mental condition, as shown in Movie S1 (Supporting Information).

To ensure the biocompatibility of the MN patch, CCK‐8 analysis and live/dead staining of L929 cells were performed. As shown in Figure 5b, cell viability of L929 cells showed no significant difference after 5 days of exposure to the MN patch. Figure S21 (Supporting Information) confirmed that no apparent dead cells were observed over this period. Additionally, the transdermal electrochemical performance of the MBFC was evaluated. The impedance measurement for 1 hour revealed that the MBFC exhibited higher internal resistance and charge‐transfer resistance compared to the in vitro tests (Figure 5c). However, the internal resistance reached approximately 5000 Ω, which was still much lower than the skin's resistance. The OCP of MBFC remained steady at around 0.28 V from 1 hour to 6 hours (Figure S22, Supporting Information). A typical insulin‐deficient diabetic mice (Glucose concentration of 18.3 mm) wearing the MBFC equipped with a 5000 Ω resistor was used to assess continuous transdermal current output (Movie S2, Supporting Information). The current initially decreased and then gradually stabilized at 4–5 µA@5 kΩ over 1‐hour to 6‐hour period, as shown in Figure 5d.

Figure S23 (Supporting Information) showed the changes of blood glucose concentration in mice wearing the insulin‐loaded MBFC, insulin‐loaded MN patch, and blank MN patch. After 6 hours, the average blood glucose concentrations of mice wearing insulin‐loaded MBFC and the insulin‐loaded MN patch were less than 7 mm (Normal blood glucose level). However, the average blood glucose concentration of mice wearing the blank MN patch still remained higher than 11.7 mm (High blood glucose level) after 6 hours. To investigate the delivery efficiency and rate of insulin, the blood glucose level and its reduction rate are shown in Figure 5e,f. The average blood glucose level and the reduction rate of glucose in mice wearing the blank MN patch remained essentially unchanged. At 1‐3 hours, the average blood glucose level and reduction rate in mice wearing the insulin‐loaded MBFC and the insulin‐loaded MN patch showed only a small difference. This phenomenon was also observed in vitro tests, in which insulin also required some time to effectively lower the blood glucose levels of the mice. After 3 hours, the average blood glucose level of mice using the insulin‐loaded MBFC was lower than that of the mice wearing the insulin‐loaded MN patch, and the reduction rate of blood glucose in the insulin‐loaded MBFC group was always faster than that of insulin‐loaded MN patch group. After 6 hours, the average blood glucose reduction was 73.6% for the insulin‐loaded MBFC group, and 59.4% for the insulin‐loaded MN patch group. These results demonstrated that the MBFC was capable of enhancing the transdermal delivery of negatively charged drugs.

Afterward, the MN patch was removed, and the back skin of the mice exhibited an array of microchannels, as shown in Figure S24 (Supporting Information), which was caused by the efficient penetration of the MN patch. Within 5 minutes, this array disappeared due to the minimal skin irritation of MBFC. Figure S25 (Supporting Information) illustrated the residue interstitial fluid on the surface of the MN patch, further demonstrating the efficient biofluid extraction directly by the MN patch. Flexible performance was crucial for wearable electronics in real‐world environments. To assess this, bending and twisting forces were applied to the breathable tape at the top of the MN patch. As shown in Figure S26 (Supporting Information), the MN patch fully adapted to the complicated deformations of the tape. Additionally, intact MN arrays were clearly observed on the patch after 6 hours, confirming that the patch could provide continuous interstitial fluid extraction and drug delivery during the operation.

### 2.6 Numerical Simulation of MBFC

**Figure 6:**
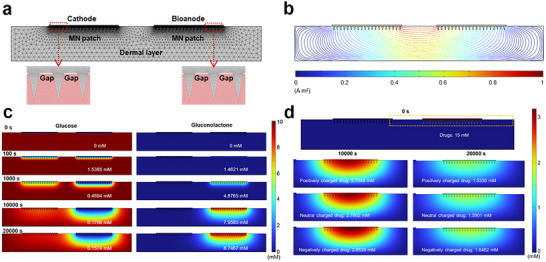
Numerical simulation of MBFC interior. a) Computational domain of the MBFC and dermal layer model. b) Schematic of electric field direction. c) Concentration distribution of glucose and gluconolactone at 0, 100, 1000, 10, 000, and 20, 000 s. d) Concentration distribution of positively charged drug, neutral charged drug, and negatively charged drug at 10, 000 and 20, 000 s. Drugs were stored into bioanode MN patch.

To clarify the mass transfer processes, a numerical simulation model was constructed, as shown in **Figure** **6**a. This model consisted of two parts: the MBFC and the dermal layer. The gaps were introduced between the MN patch substrate and the dermal layer to ensure that glucose, GDL, and drugs penetrate through the subcutaneous layer via the MNs, rather than through the corneum. The geometric parameters of the model and detailed physical parameters were listed in Tables S2 and S3 (Supporting Information). Figure 6b illustrated the electric field direction within this model. It showed the positive charges were more easily transferred from the bioanode to the cathode, and negative charges more easily moved from the cathode to the bioanode. Electroosmotic flow direction (Figure S27, Supporting Information) indicated that drugs tended to migrate from the bioanode to the cathode. However, the influence of the electric field was far more significant than that of electroosmotic flow, and it played a dominant role in drug delivery. This result supported the experimental results that drugs were more easily delivered when positively charged drugs were positioned interior the bioanode, and negatively charged drugs interior the cathode.

Furthermore, Figure 6c and Figure S28a (Supporting Information) illustrated the concentration distribution of glucose and GDL within the model, as well as the average concentration of these substances in the MN patch near the bioanode. Initially, the concentrations of glucose in the interstitial fluid and MN patches were 10 and 0 mm, respectively. Then, glucose gradually diffused into the MN patches. Since glucose was not involved in reactions at the cathode, the glucose concentration near the cathode stabilized at 10 mm. In contrast, glucose was consumed at the bioanode, which led to continuous consumption and replenishment in the patch near the bioanode. The glucose concentrations at 100, 1000, 10 000, and 20 000 s were shown in Figure 6c. Near the bioanode, the concentration initially increased, then decreased as the reaction progressed. This decrease in glucose concentration was attributed to the slow diffusion of glucose and its rapid consumption at the bioanode.

The current density at the bioanode also followed a similar trend (Figure S28b, Supporting Information). The current density at the bioanode also followed a similar trend, which increased initally and then decreased in response to the glucose concentration changes. After 20 000 s, the current density at the bioanode stabilized at ≈7 µA cm^−2^, which was consistent with the experimental results. Meanwhile, GDL continued to form on the electrode surface and was metabolized by the body. However, the GDL concentration near the bioanode continued to rise and accumulated as the reaction proceeded.

Furthermore, positively charged, negatively charged, and neutral charged drugs were loaded into the MN patch near the bioanode to simulate their delivery processes, as shown in Figure 6d. Similar to previous results, the drugs were delivered into the dermal layer, and the drug concentration in the MN patch continued to decrease. A low drug concentration in the patch indicated that drug delivery was enhanced over time. As shown in Figure 6d and Figure S28a (Supporting Information), the concentration of the positively charged drug near the bioanode was the lowest, while the concentration of the negatively charged drug was the highest throughout the process. It demonstrated that the delivery of positively charged drugs was enhanced and the delivery of negatively charged drugs was inhibited. As comparably, the drugs were stored in the MN patch near the cathode (Figure S29, Supporting Information). The delivery of positively charged drugs was inhibited and the delivery of negatively charged drugs was enhanced. Combining the experimental and computational results, the drug releasing performance is significantly increased with the BFC integrated MN patch.

## 3 Conclusion 

In the study, we took advantage of MN patches to fabricate the MXene‐based BFC by utilizing the interstitial fluid. The MN patch possess the properties of high porosity of 93.5%, well failure stress of 5 N needle^−1^ and rapid swelling of ≈1168.4% for 1 h, which enabled both the transdermal extraction of glucose as biofuel, and transdermal drug delivery. The stable transdermal current of 4–6 µA@5 kΩ was collected for 6 hours in vitro and in vivo. The existence of external electrical field could facilitate the transdermal delivery of drugs with varying molecular weights and charges. As a typical cation drug, the CIP‐loaded MBFC achieved a cumulative ciprofloxacin delivery of 13.5%, which represented a ≈30% increasing compared to the conventional CIP‐loaded MN patch. The insulin served as the anion trial to reduce the blood glucose levels in insulin‐deficient diabetic mice. The average blood glucose reduction was 73.6% for the insulin‐loaded MBFC, and 59.4% for the conventional insulin‐loaded MN patch. The theoretical simulations revealed the good drugs transdermal delivery to fast electron transport and substrate adsorption provided by MXene‐based bioanode, continuous interstitial fluid extraction, and stable current output provided by MN patch. The MBFC held great potential for biomedical applications in wearable electronics and transdermal drug delivery.

## 4 Statistical Analysis

Statistical analysis was calculated using the program Microsoft Excel. Both the measurement errors of current and potential are 0.2% for CHI 760e electrochemical workstation. The sample size (*n*) for each statistical analysis is denoted in the corresponding figure caption, and the evaluation of the CIP delivery (Figure 4f), CCK‐8 analysis of microneedle patch (Figure 5b) and the mice blood glucose (Figure 5e,f; Figure S21, Supporting Information) were repeated and expressed as means ± standard deviation (SD).

## Conflict of Interest

The authors declare no conflict of interest.

## Author Contributions

Y.Y., Q.F., and Q.L. conceived and supervised this research. S.G. designed and carried out experiments and characteristics. Q.F., J.W., and J.Z. carried out biological experiment. R.C. and D.Y. analyzed the data. X.Z. and Q.L. revised the paper. All authors discussed the results and commented on the paper.

## Supporting information



Supporting Information

Supporting Information

Supporting Information

## Data Availability

The data that support the findings of this study are available from the corresponding author upon reasonable request.
