# Supplementary material for: Microneedle‐Based Biofuel Cell with MXene/CNT Hybrid Bioanode: Fundamental and Biomedical Application
Source: Adv Sci (Weinh). 2025 Sep 29;12(46):e16229. doi: 10.1002/advs.202516229 (PMC12697762; doi:10.1002/advs.202516229)
Supplement: Supplementary file 1 — Supporting Information [file ADVS-12-e16229-s003.docx]

***Supporting Information***

**Microneedle-Based Biofuel Cell with MXene/CNT Hybrid Bioanode: Fundamental and Biomedical Application**

Shoujie Guan^1,2^, Jingxi Wang^3^, Yang Yang^1,2*^, Xun Zhu^1,2^, Jianping Zhou^3^, Dingding Ye^1,2^, Rong Chen^1,2^, Qinlin Fan^4*^, Qiang Liao^1,2*^

^1^ Key Laboratory of Low-grade Energy Utilization Technologies and Systems, Chongqing University, Ministry of Education, Chongqing 400030, China

^2^ Institute of Engineering Thermophysics, School of Energy and Power Engineering, Chongqing University, Chongqing 400030, China

^3^ Department of Orthodontics, The affiliated Hospital of Stomatology, Chongqing Medical University, Chongqing 400030, China

^4^ Department of Neurology, Second Affiliated Hospital of Army Medical University, Chongqing 400030, China

E-mail: [yang_yang@cqu.edu.cn](mailto:yang_yang@cqu.edu.cn) (Y. Yang).

**1 Experimental**

1. **Fabrication of MXene/CNT/GOx bioanode**

To fabricate the MXene/CNT/GOx bioanode, 2 mg of PDDA solution (20 wt%, Average molecular weight: 4 000 000–6 000 000, Sigma-Aldrich Trading Co., Ltd., Shanghai, China) was firstly dispersed in 1 mL of single-walled CNT aqueous solution (4 mg mL^-1^, Diameter: 1-2 nm, Length: 10-30 μm, Jiacai Technology Co., Ltd., Chengdu, China) to obtain CNT-PDDA suspension. Then, 0.3 mL of CNT-PDDA suspension and 2 mL of single-layer MXene solution (5 mg mL^-1^, Size: 0.2-1 μm, Electric conductivity: 3500±500 S cm^-1^, Jiacai Technology Co., Ltd., Chengdu, China) were mixed evenly to obtain the MXene/CNT suspension labeled as solution A. The single-layer MXene was obtained by etching Ti_3_AlC_2_ and adding the tetramethylammonium hydroxide as intercalate reagent. Furthermore, 50 μL of 0.2 M 1,4-NQ (Tianjin Heowns Biochemical Technology Co., Ltd., Tianjin, China) solution dissolved in ethanol (Shanghai Macklin Biochemical Co., Ltd., Shanghai, China) and acetone (Chengdu Kelong Chemical Co.,Ltd., Chengdu, China) (9:1, V/V) was labeled as solution B. 50 μL of glucose oxidase (GOx, 40 mg mL^−1^, 100 U mg^-1^, Shanghai Macklin Biochemical Co., Ltd., Shanghai, China) dispersed in 0.1 M phosphate buffer saline (PBS, Shanghai Macklin Biochemical Co., Ltd., Shanghai China) was labeled as solution C. 30 μL of 1% glutaraldehyde (Innochem Technology Co., Ltd., Beijing, China) aqueous solution was labeled as solution D1, 30 μL of 1 wt% chitosan (Beijing Jinming Biochemical Technol. Co., Ltd., Beijing, China) in 0.1 M acetic acid (Chengdu Keshi Technology Co., Ltd., Chengdu, China) was labeled as solution D2. Solution A, solution B, solution C, the mixture of solution D1 and D2 were dripped onto the buckypaper (1 cm^2^, Jiacai Technology Co., Ltd., Chengdu, China) continually to prepare the MXene/CNT/GOx bioanode. One drop step was performed once the previous dropped solution was completely dried at room temperature.

1. **Fabrication of methacryloylated hyaluronic acid (HAMA) microneedle patch**

As photo-initiator, 25 mg Lithium Phenyl (2,4,6-trimethylbenzoyl) phosphinate (LAP, EFL-Tech Co., Ltd., Suzhou, China) was dissolved in 10 mL of 0.1 M PBS solution at 45 °C. Then, 0.5 g of HAMA precursor (EFL-Tech Co., Ltd., Suzhou, China) and drug were dissolved in the above solution in sequence to form the precursor solution. After that, the precursor solution was dropped into MN patch mold (Xiamen Qiepu Medical Technology Co., Ltd., Xiamen, China) based on the PDMS and deformed at vacuum state, then concentrated until it was completely dry at 30 °C. Finally, the drug-loaded MN patch (Weight: 60~70 mg per patch) was obtained at ultraviolet light (Wavelength: 405 nm) for 5 s and demolding. The drug-loaded MN patch was fabricated including CIP-loaded MN patch with a mass loading of 5.7 mg CIP per patch (Beijing Dehangwuzhou Technology Co., Ltd., Beijing, China), and insulin-loaded MN patch with a mass loading of ~11.2 mg insulin per patch (Beijing Solarbio Technology Co., Ltd., Beijing, China). Comparably, the blank MN patch (50 mg per patch) was prepared without the addition of drug using the same preparation technique.

1. **Fabrication of microneedle-based biofuel cell**

The microneedle-based biofuel cell was composed of a MXene/CNT/GOx bioanode, a Pt/C cathode and two MN patches. Biomedical glue was coated to the edges of the cathode and bioanode, which were separately affixed onto a MN patch surface (Surface without MNs). A 5000 Ω resistor wire was glued in bioanode surface and cathode surface (Surface without catalysts) using conductive silver paste to allow the electronic transmission. Finally, bioanode surface and cathode surface (Surface without catalysts) were further affixed onto a medical breathable tape. The distance between bioanode and cathode were set at 5 mm to guarantee the safe operation during the wearable environment and maintain a good electrochemical performance.

1. **Materials characterizations**

The structures and morphologies of electrodes and hydrogel-based MN patch were investigated using the field-emission scanning electron microscopy (SEM, SU8020, Hitachi, Japan) equipped with an energy dispersive X-ray spectroscopy (EDX) detector, and field-emission transmission electron microscopy (TEM, Tecnai G2 F30 S-TWIN, FEI, OSU). To observe the distribution of GOx, it was labeled by fluorescein isothiocyanate (FITC, Thermo Fisher Scientific Inc., USA) and studied using the confocal laser scanning microscopy (CLSM, TCS SP8 CSU, Leica, Germany). The composition species and characteristic functional groups of bioanode were determined using X-ray diffractometer (XRD, Cu Kα, λ=0.1541 nm, D8 ADCANCR, BRUCKER, Germany), X-ray photoelectron spectroscopy (XPS, Thermo Fisher Escalab 250Xi XPS spectrometer, MA, Al Kα) and Fourier Transform Infrared Spectrometer (FTIR, Thermo Nicolet IS10, MA). Of which, XRD was also used to determine the interlayer spacings of MXene and MXene/CNT, as calculated using Bragg's equation:

$$2dSin\theta=n\lambda$$

In the equation, *d* denoted the lattice interplanar spacing of the crystal, $\theta$ represented the X-ray incidence angle and $\lambda$ denoted the wavelength of the characteristic X-rays.

The Zeta potentials of materials and CIP were measured using a Zetasizer Nano ZS90 (Malvern Panalytical Ltd., UK). The failure stress of MN patch was studied using texture analyzer (Shanghai Prosun Industrial Development Co., Ltd., China). The average pore diameter and porosity of full swelling MN patch were measured using the mercury porosimetry (AutoPore V9620, Monitor instrument Co., Ltd., USA). The swelling rate of HAMA MN patch was characterized by calculating the percentage of weight increment (Soaking in 0.1 M PBS) at special time and weight of dry MN patch.

1. **Electrochemical measurements**

To characterize the electrochemical properties of electrodes and biofuel cells, electrochemical impedance spectroscopy (EIS) and LSV were collected on the CHI 760e and VMP3 multichannel electrochemical workstations. The electrochemical properties of bioanode and cathode were carried out using the conventional three-electrode setup with the MXene/CNT/GOx bioanode or Pt/C cathode as working electrode, Ag/AgCl [0.198 V *vs.* standard hydrogen electrode (SHE))] as reference electrode, platinum plate or graphite rod as counter electrode. Particularly, the ORR performance of Pt/C catalyst powders was tested using the rotating ring-disk electrode (RRDE) with the rotation speed of 1600 rpm. The power density plots of biofuel cells were obtained *via* LSV procedure, which was recorded from OCP to 0.01 V with a scan rate of 10 mV s^-1^ in 0.1 M PBS containing 10 mM glucose.

1. **In vitro transdermal electrochemical and drug delivery performance**

To characterize the transdermal performance, MBFC was applied to the skin of a Bama miniature pig (Taizhou Taihe Biotechnology Co., Ltd., Taizhou, China) with an external resistance of 5000 Ω resistor. The CIP was loaded into the MN patch positioned interior the bioanode. Before the test, the MBFC was pressed into the skin for 10 seconds and immobilized using medical breathable tape. The skin was then transferred to a Franz Diffusion Cell (FDC) system (Henan Yichi Trading Co., Ltd., Luoyang, China). The transdermal electrochemical performance and drug concentration were measured using an electrochemical workstation and ultraviolet spectroscopy (ZW-1712, Beijing Puxi General Instrument Co., Ltd., China).

1. **In vivo electrochemical and drug delivery performance**

The insulin-deficient diabetic mice model was established using a standard method. ^[1, 2]^ The insulin was loaded into the MN patch positioned interior the cathode. The healthy mice (10-11 weeks of age, License number: SCXK 2022-0011) were approved by the *Laboratory Animal Welfare and Ethics Committee* of the Army Medical University (License number: SYXK 2022-0018). All the experiments were complied with the ARRIVE guidelines, and under the approval of the Ethics Committee of the Stomatological Hospital of Chongqing Medical University (Protocol number: 2024.173) and Laboratory Animal Welfare and Ethics Committee Of the Army Medical University (Protocol number: AMUWEC20245290). The mice were fasted for 12 hours before receiving an intraperitoneal injection of streptozotocin (STZ, 60 mg/kg, Abmole Bioscience Inc., Houston, USA) in citrate buffer (pH: 4.5). After a week of continuous injections, the diabetic mice model was successfully established. The set-up of MBFC was similar with the in vitro measurement. The blood glucose concentrations of the mice were monitored using a glucometer (Sinocare Inc., Changsha, China) to indirectly assess the insulin delivery rate and efficiency.

1. **DFT calculations**

DFT calculations were performed on the CNT model, MXene model and MXene/CNT model using the Dmol3 program with the GGA-PBE functional. The core electrons were treated explicitly as all-electron systems, incorporating relativistic effects. The DNP 4.4 basis set was employed, with an orbital cutoff of 5.1 Å. Long-range van der Waals interactions were corrected using Grimme’s D2 method. The convergence criterion for the self-consistent field (SCF) calculation was set to 1.0 × 10^-6^ eV. For geometry optimization, the convergence criteria were set to 1.0 × 10^-5^ Ha for the maximum energy change, 0.002 Ha/Å for the maximum force, and 0.005 Å for the maximum displacement. The adsorption energy (*E_a_*) was calculated using the following formula:

$$Ea=E(X/host)-E(X)-E(host)$$

Where X represented glucose, and host referred to CNT, MXene or MXene/CNT.

1. **Numerical model**
2. Model assumption

The model was established with the following assumptions:

1) Electrochemical reactions occurred exclusively on the electrode surface without considering the electrode thickness;

2) The oxygen concentration at the electrode surface was equal to that of ambient atmosphere;

3) The pore distribution within the MN patch was uniform;

4) The dermal layer was treated as a homogeneous porous medium without considering the convective flow of interstitial fluid within the dermal layer;

5) The properties of the interstitial fluid within the dermal layer were similar to the drug solution inside the MN patches.

6) The volume remained stably interior the MN patches during the drug delivery process;

7) The impact of electrical stimulation on the physical property of dermal layer was not considered.

8) The metabolism of gluconolactone was not taken into account interior the dermal layer.

1. Mass transport

To further elucidate the mass-transfer and electrochemical reaction behaviors, a two-dimensional MBFC model was established. In terms of the mass transport behaviors, it was primarily considered as three manners of diffusion, electromigration and electroosmotic flow (*i.e*. electroosmotic drag). The governing differential equation was showed as following:

$\frac{\partial\left(\varepsilon C_{i}\right)}{\partial t}+\nabla\cdot J_{i}+u\cdot\nabla C_{i}=R_{i}$ (1)

In the equation, *C_i_* denoted the concentration of species *i*, *ε* represented the porosity of the computational domain. *R_i_* was the reaction source term, which was presented at the electrode surface. ***u*** denoted the equivalent velocity of electroosmotic flow, while ***J****_i_* represented the transport flux, encompassing both diffusion flux and the flux due to the electric field effect. The specific forms were as follows:

 (2)

Then, *z_i_* denoted the charge number of species *i*. *D_i_^eff^* represented the effective diffusion coefficient, *F* was the Faraday constant, and *φ* signified the potential distribution within the computational domain. *u_im,i_* corresponded to the ionic mobility, which was determined by the Nernst-Einstein relation:

$u_{im,i}=\frac{D_{i}^{eff}}{RT}$ (3)

*R* denoted the universal gas constant, and *T* represented the thermodynamic temperature. The effective diffusion coefficient within the MN patch and dermal layers was calculated using the following relationship:

$D_{i}^{eff}=\frac{\varepsilon}{\tau}D_{i}$ (4)

Where, *D_i_* represented the diffusion coefficient of species *i* in the liquid phase. *τ* denoted the tortuosity of the computational domain, which was calculated using the Millington-Quirk model:

$\tau=\varepsilon^{-\frac{1}{3}}$ (5)

In Equation (1), the distribution of the equivalent electroosmotic drag velocity was determined by the following expression:

$\nabla\cdot u=0$ (6)

 (7)

Where, *ε_0_* represented the dielectric constant of vacuum, *ε_w_* denoted the relative dielectric constant of this system, *ζ* represented the zeta potential, and *μ* was the dynamic viscosity of the fluid.

1. Conservation of charge and electrochemical reaction

Electric potential distribution in the dermal layer and porous MN patch was governed by the following relationship:

 (8)

 (9)

In this expression, *σ_dl_^eff^* and *σ_mn_^eff^* denoted the effective conductivities of the dermal layer and MN patches, respectively, while *φ_dl_* and *φ_mn_* represented the local electric potential in the dermal layer and MN patches.

The current densities at the bioanode and cathode were determined by the Butler-Volmer equation:

$\text{i}_{\text{a}}=\text{i}_{\text{0,g}}\left(\frac{C_{g}}{C_{\text{g,ref}}}\right)^{\beta}\left[\exp\left(\frac{\alpha_{\text{a}}\text{F}}{\text{RT}}\eta_{\text{a}}\right)-\exp\left(\frac{-\alpha_{\text{c}}\text{F}}{\text{RT}}\eta_{\text{a}}\right)\right]$ (10)

$\text{i}_{\text{c}}=\text{i}_{\text{0,O}}\left(\frac{C_{\text{O}}}{C_{\text{O,ref}}}\right)^{\beta}\left[\exp\left(\frac{\alpha_{\text{a}}\text{F}}{\text{RT}}\eta_{\text{c}}\right)-\exp\left(\frac{-\alpha_{\text{c}}\text{F}}{\text{RT}}\eta_{\text{c}}\right)\right]$ (11)

Here, *i_0,g_* represented the bioanode exchange current density, *c_g_* was the local glucose (Biofuel) concentration, *C_g,ref_* denoted the reference glucose concentration, and *β* was the reaction order. *α_a_* and *α_c_* were the charge transfer coefficients. *i_0,O_* represented the cathode exchange current density, *C_O_* was the local oxygen concentration, and *C_O,ref_* was the reference oxygen concentration. *η_a_* and *η_c_* were the overpotentials at the bioanode and cathode, respectively. The expressions of *η* were as follows:

$\eta=\varphi_{s}-\varphi_{mn}-E_{eq}$ (12)

In which, *E_eq_* represented the electrode equilibrium potential, *φ_s_* denoted the electrode potential.

The electrochemical reaction source term was determined using Faraday's law:

$R_{g}=-\frac{\text{v}_{\text{g}}\text{i}_{\text{a}}}{\text{n}_{\text{g}}\text{F}}$ (13)

Where, *n_g_* was the number of electrons involved in the glucose reaction, and *v_g_* denoted the stoichiometric coefficient.

The parameters used in the model calculations were provided in **Table S2** and **Table S3**.

(4) Boundary and initial conditions

The boundary and initial conditions were shown in **Fig. S30**. With respect to mass transport, the left, right and bottom surfaces of the dermal layer were maintained at constant environmental concentrations. Specifically, drug and GDL concentrations were set to zero, and glucose concentration was set to 10 mM. The interface between MN and dermal layer was governed by continuity boundary condition, ensuring the consistent flux across the contact surface. All other external boundaries were subject to no-flux conditions. For the electric potential distribution, Dirichlet boundary conditions were applied at the bioanode and cathode surfaces, their potentials were set to 0 V and 0.05 V, respectively. The remaining external boundaries were treated as electrically insulated to eliminate any potential flux through these regions.

At the beginning of the simulation, the potential across all locations within the computational domain was set to 0 V. The glucose concentration in the dermal layer was 10 mM, while the concentrations of the drug and GDL were both 0. In the MN patch near the bioanode or cathode, the drugs concentrations were 15 mM. However, in the MN patch near the cathode or bioanode, the drugs concentrations were also 0 mM. Throughout all MN patch, regardless of location, the concentrations of glucose and GDL were both 0.

**2 Supplementary Figures**


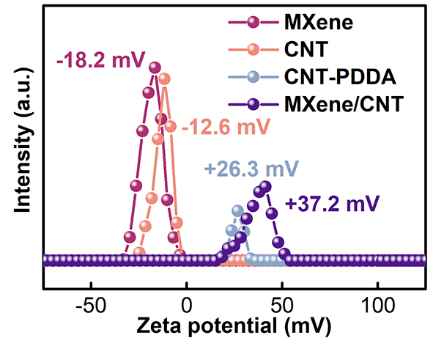


**Fig. S1** The Zeta potentials of MXene, CNT, CNT-PDDA and MXene/CNT.


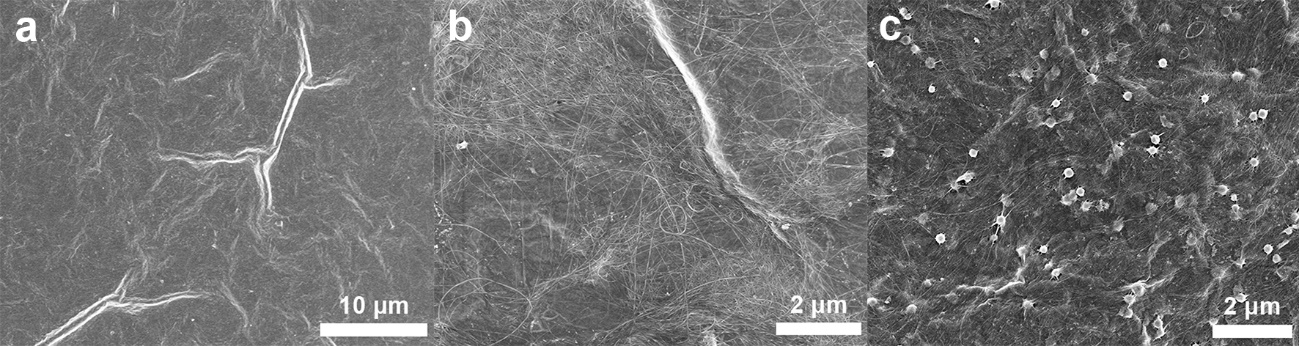


**Fig. S2** The SEM images of MXene **a)**, MXene/CNT **b)** and MXene/CNT/GOx **c)**.


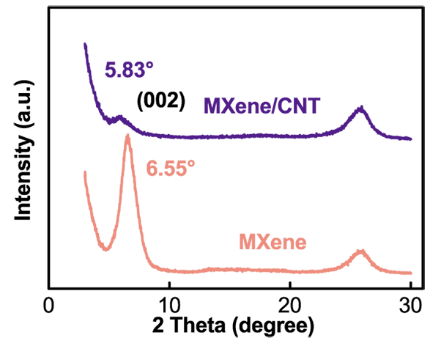


**Fig. S3** XRD patterns of MXene and MXene/CNT.


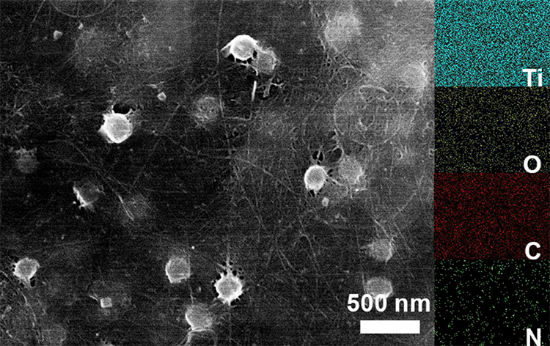


**Fig. S4** The SEM image of MXene/CNT/GOx and the corresponding EDS mapping.


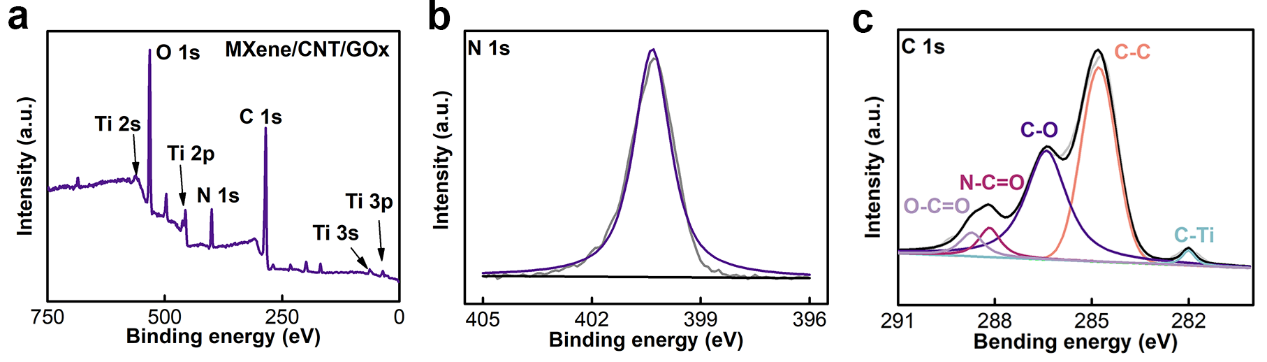


**Fig. S5 a)** XPS spectrum of MXene/CNT/GOx bioanode. High-resolution XPS spectra of N 1s **b)** and C1s **c)** of MXene/CNT/GOx bioanode.


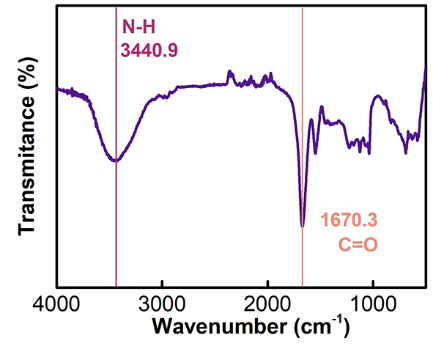


**Fig. S6** FTIR spectroscopy of MXene/CNT/GOx bioanode.


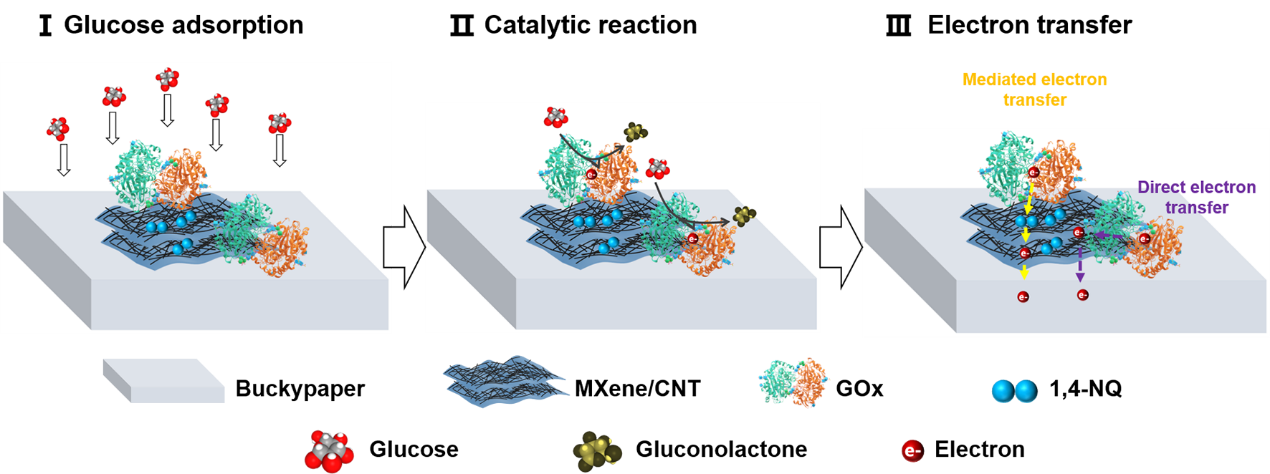


**Fig. S7** Schematic diagram of catalytic reaction process of MXene/CNT/GOx for BFCs.


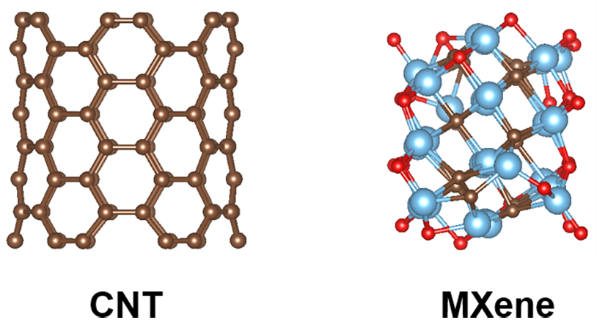


**Fig. S8** Optimized crystal structure of CNT and MXene.


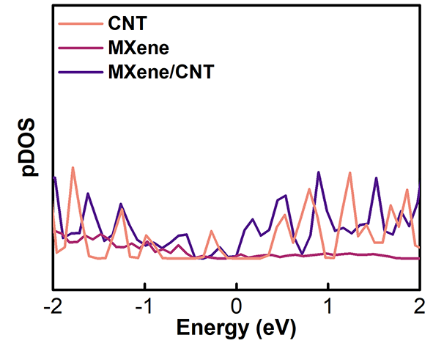


**Fig. S9** The electron density of states of CNT, MXene and MXene/CNT for P-orbital (pDOS).


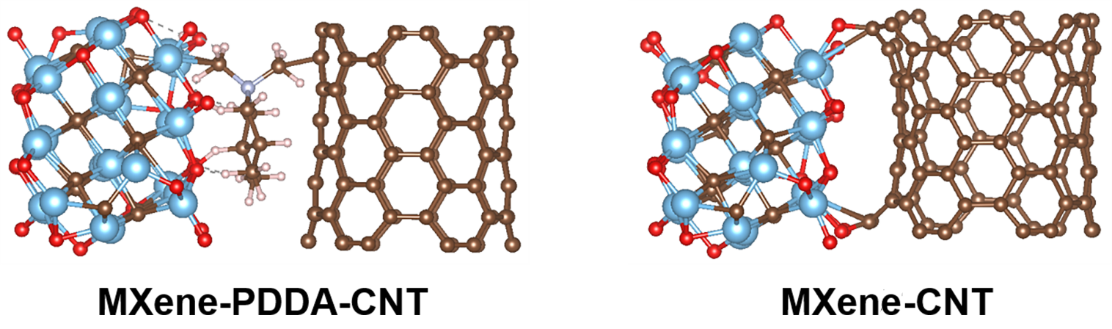


**Fig. S10** Optimized crystal structure of MXene-PDDA-CNT and MXene-CNT.


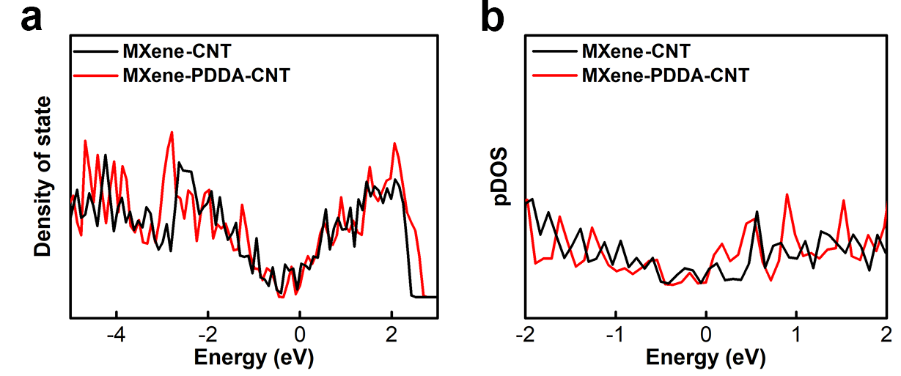


**Fig. S11** DOS **a)** and pDOS **b)** for MXene-PDDA-CNT and MXene-CNT.


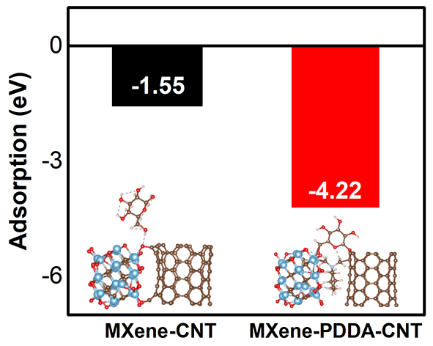


**Fig. S12** Adsorption energies of glucose adsorbed on the lattice of MXene-PDDA-CNT and MXene-CNT. Inset photos were the corresponding adsorption optimized structures.


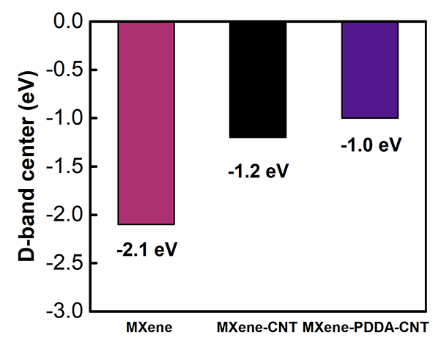


**Fig. S13** D-band center for MXene, MXene-PDDA-CNT and MXene-CNT.


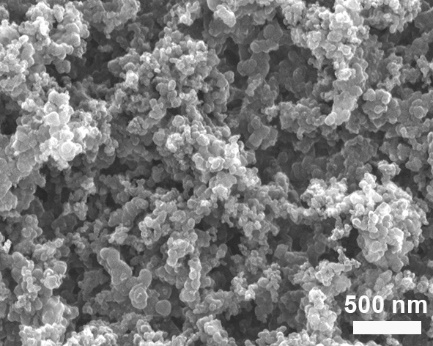


**Fig. S14** The SEM image of Pt/C.


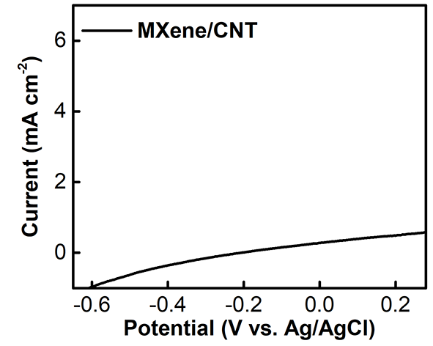


**Fig. S15** LSV curve of MXene/CNT in 0.1 M PBS containing 10 mM glucose.


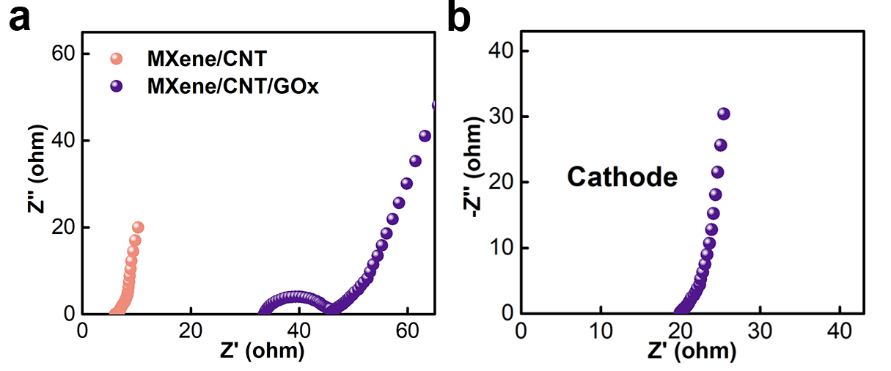


**Fig. S16** Nyquist plots of MXene/CNT and MXene/CNT/GOx bioanode **a**) and Pt/C cathode **b)** in 0.1 M PBS containing 10 mM glucose using conventional three-electrode setup.


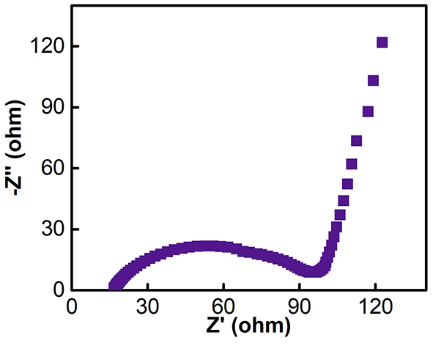


**Fig. S17** Nyquist plots of BFC composed of MXene/CNT/GOx bioanode and Pt/C cathode in 0.1 M PBS containing 10 mM glucose using conventional two-electrode setup.


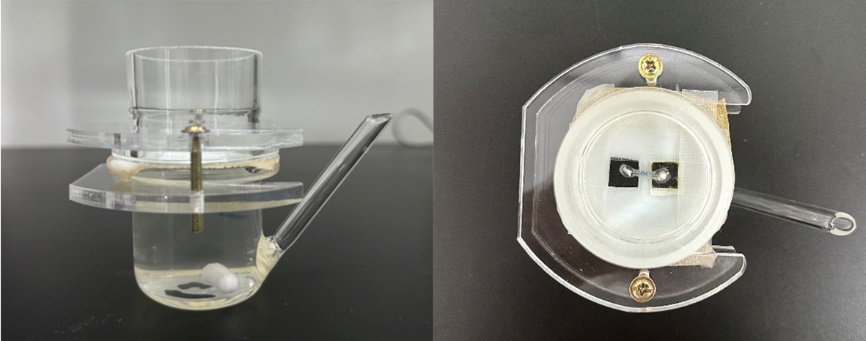


**Fig. S18** The photos of FDC system equipped with MBFC and the skin of a Bama miniature pig.


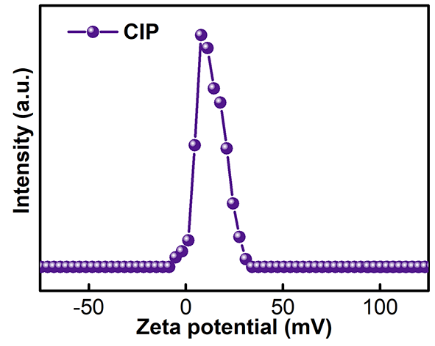


**Fig. S19** The Zeta potential of CIP aqueous solution.


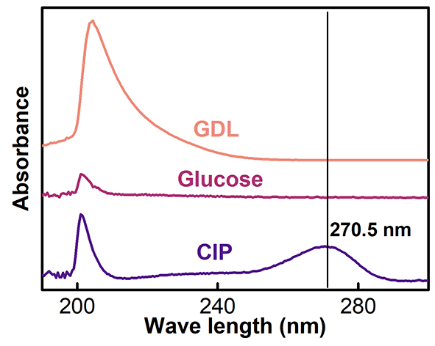


**Fig. S20** The ultraviolet spectroscopy of GDL, glucose and CIP.


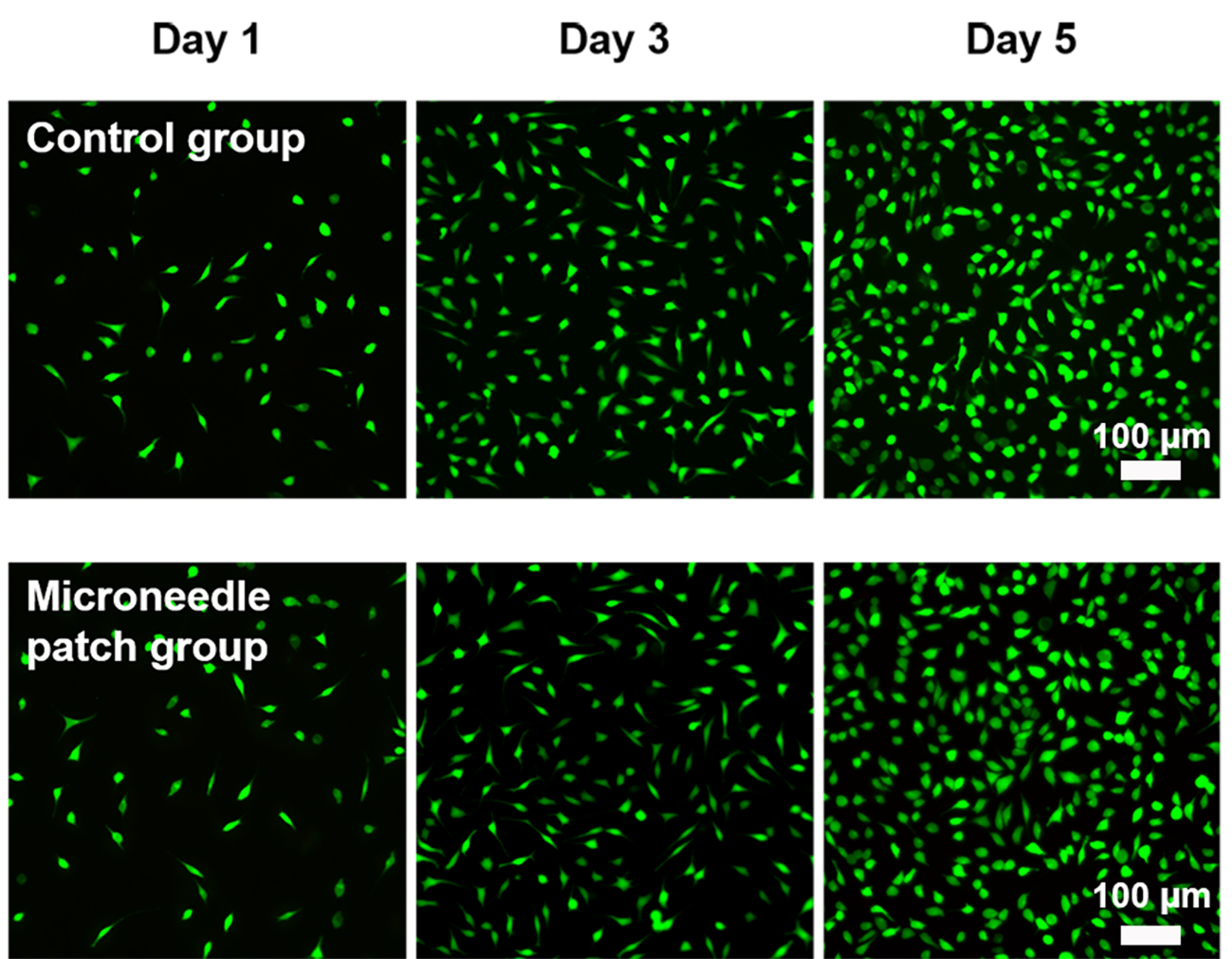


**Fig. S21** Live/dead staining images of L929 cells in control group and MN patch group on days 1, day 3 and day 5.


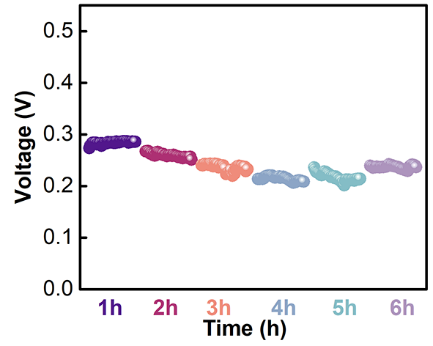


**Fig. S22** The OCP of MBFC in vivo, a typical 50 second of data was recorded every hour.


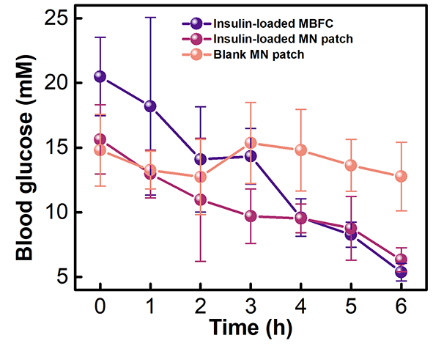


**Fig. S23** The blood glucose concentration of mice wearing different patches for 6h (n=3).


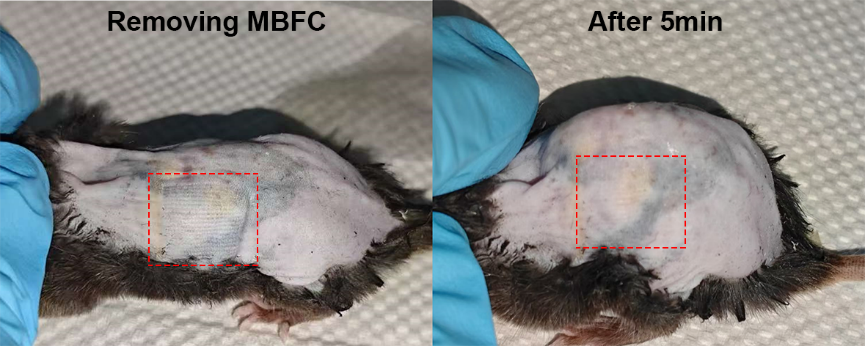


**Fig. S24** These photos of mice removing MBFC**.**


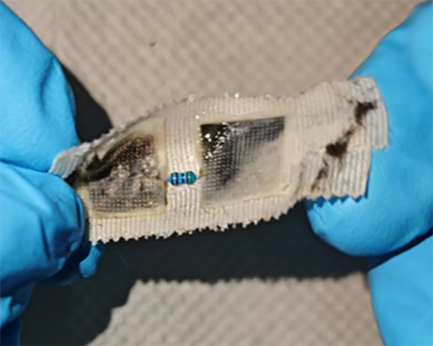


**Fig. S25** The photo of MBFC after 6 hours of operation.


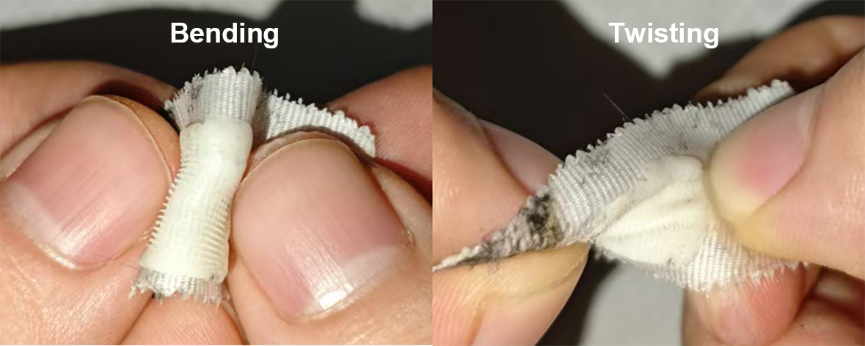


**Fig. S26** These photos of MN patch enduring bending and twisting after 6 hours of operation.


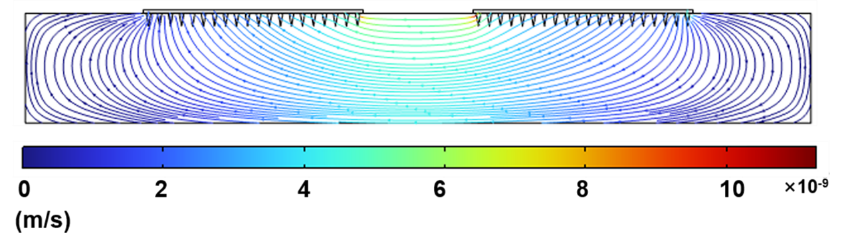


**Fig. S27** Schematic of electroosmotic flow direction.


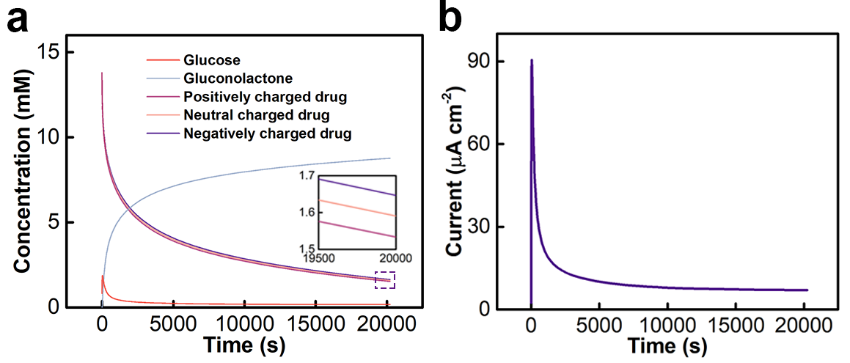


**Fig. S28 a)** The computational concentrations of glucose, gluconolactone, positively charged drug, neutral charged drug and negatively charged drug in MN patch positioned interior bioanode over 20000 s. **b)** The computational average current density at the bioanode surface over 20000 s.


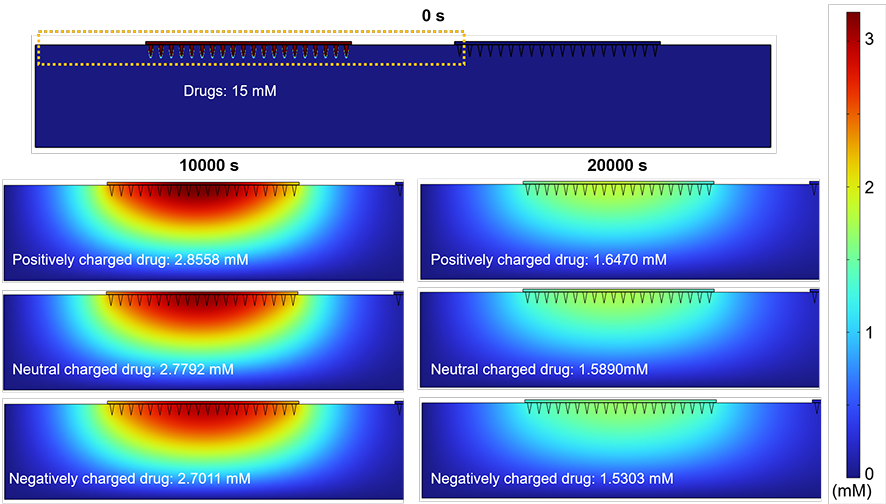


**Fig. S29** Concentration distribution of positively charged drug, neutral charged drug and negatively charged drug in the model for 10000 s and 20000 s. Drugs were stored into cathode MN patch.

**
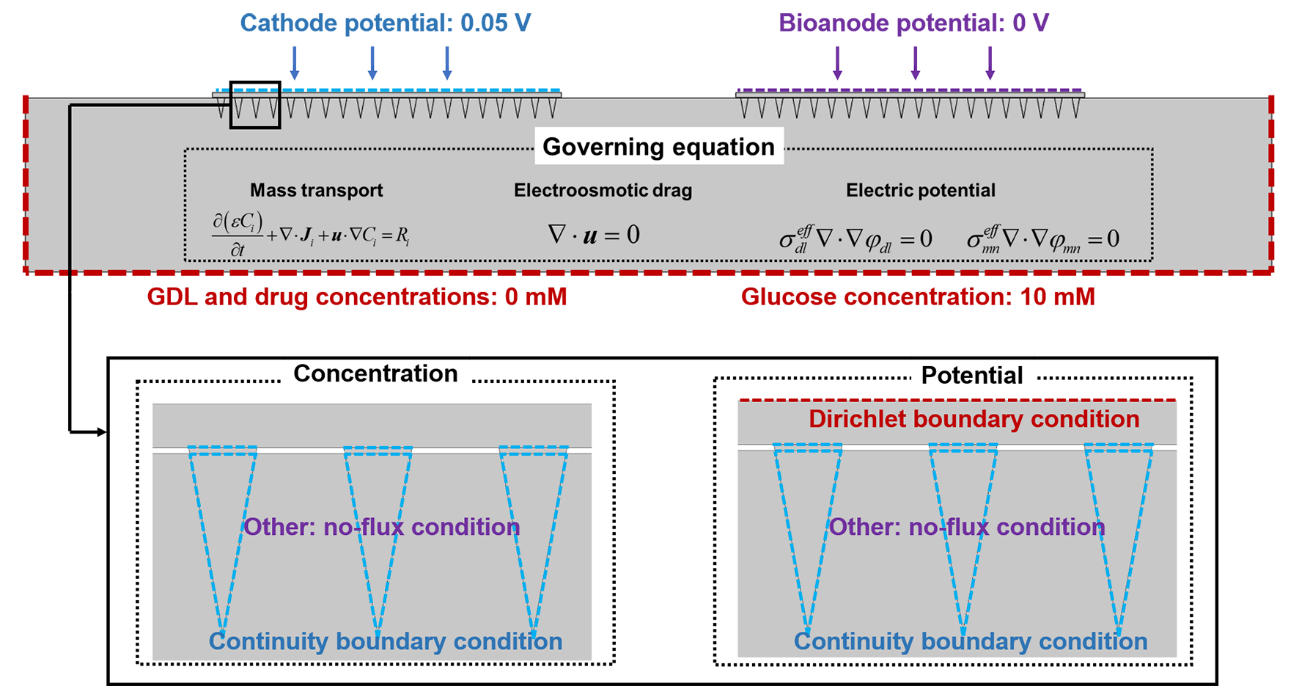
**

**Fig. S30** Governing equations, boundary and initial conditions of numerical simulation

**3 Supplementary Tables**

**Table S1** Bader charge results before and after introducing PDDA.

|  | Before introducing PDDA | After introducing PDDA |
| --- | --- | --- |
| C-PDDA | 1.307 eV | -9.370 eV |
| N-PDDA | -0.747 eV | -0.708 eV |
| Ti-MXene | -0.559 eV | -1.641 eV |
| Ti-MXene | 48.153 eV | 47.003 eV |
| C-MXene/CNT | -20.036 eV | -16.213 eV |
| O-MXene | -27.005 eV | -23.062 eV |

**Table S2** Geometric parameters of MBFC and dermal layer model.

| Parameter | Value |
| --- | --- |
| Cathode length, *L_c_* | 10 mm |
| Anode length, *L_a_* | 10 mm |
| MN patch substrate thickness, *W_mn_* | 0.14 mm |
| Thickness of the dermal layer, *W_dl_* | 4.98 mm |
| Length of the dermal layer, *L_dl_* | 35.73 mm |
| Diameter of the MNs, *d_mn_*  Height of the MNs, *h_mn_*  Distance between the tips of two MNs, *d_mn-mn_* | 0.22 mm  0.6 mm  0.5 mm |
| Number of MNs, *n_mn_* | 20 |
| Distance between the MN patch and dermal layer, *d_mn-dl_* | 0.02 mm |

**Table S3** Model calculation parameters of MBFC and dermal layer model.

| Parameter | Value | Reference |
| --- | --- | --- |
| Glucose diffusion coefficient, *D_g_* | 2×10^-9^ m^2^ s^-1^ | [3] |
| Gluconolactone diffusion coefficient, *D_gl_* | 2×10^-9^ m^2^ s^-1^ | Assumed |
| Drugs diffusion coefficient, *D_dp_*, *D_dn_*, *D_d0_* | 1×10^-9^ m^2^ s^-1^ | Assumed |
| Porosity of the dermal layer, *ε_dl_* | 0.2 | [4] |
| Porosity of the MN patch, *ε_mn_* | 0.9 | Experiment |
| Reference glucose concentration, *C_g,ref_* | 10 mM | Assumed |
| Reference oxygen concentration, *C_O,ref_* | 8.5 mol m^-3^ | [5] |
| Reaction order, *β* | 1 | [5] |
| Effective conductivities of dermal layer, *σ_dl_^eff^* | 0.434 S m^-1^ | [6] |
| Effective conductivities of MN patch, *σ_mn_^eff^* | 0.832 S m^-1^ | Experiment |
| Faraday constant, $\text{F}$ | 96485 C mol^-1^ |  |
| Universal gas constant, $\text{R}$ | 8.314 J mol^-1^ K^-1^ |  |
| Operation temperature, $\text{T}$ | 298 K |  |
| Charge transfer coefficient, *α_a_* and *α_c_* | 0.5 | ^3^ |
| Cathode equilibrium potential, *E_eq,c_* | 1.23 V |  |
| Anode equilibrium potential, *E_eq,a_* | 0.051 |  |
| Cathode exchange current density, *i_0,O_* | 0.001 A m^-2^ | Assumed |
| Anode exchange current density, *i_0,g_* | 0.001 A m^-2^ | Assumed |
| Zeta potential, *ζ* | -0.1 V | [7] |
| Dynamic viscosity, *μ* | 0.001 Pa s | [7] |
| Dielectric constant of vacuum, *ε_0_* | 8.854×10^-12^ F m^-1^ |  |
| Relative dielectric constant, *ε_w_* | 20 | [8] |

**References**

[1] C. Yang, T. Sheng, W. Hou, J. Zhang, L. Cheng, H. Wang, W. Liu, S. Wang, X. Yu, Y. Zhang, J. Yu, Z. Gu, *Sci. Adv.* **2022**, 8, eadd3197.

[2] D. Maity, P. Guha Ray, P. Buchmann, M. Mansouri, M. Fussenegger, *Adv. Mater.* **2023**, 35, e2300890.

[3] E. Khalil, K. Kretsos, G. B. Kasting, *Pharm. Res.* **2006**, 23, 1227-1234.

[4] P. Liu, T. Zhang, Y. Huang, *Front. Bioeng. Biotechnol.* **2024**, 12, 1347159.

[5] B. Zhang, D.-d. Ye, P.-C. Sui, N. Djilali, X. Zhu, *J. Power Sources* **2014**, 259, 15-24.

[6] K. Wake, K. Sasaki, S. Watanabe, *Phys. Med. Biol.* **2016**, 61, 4376-4389.

[7] M.-S. Chun, *Korean J. Chem. Eng.* **2022**, 19, 729.

[8] S. A. R. Naqvi, M. Manoufali, B. Mohammed, A. T. Mobashsher, D. Foong, A. M. Abbosh, *IEEE Trans. Instrum. Meas.* **2021**, 70, 1-10.
